# Continuous Light‐Induced Water Oxidation in Polyoxometalate‐Based Photocatalytic Protocells and Prototissues

**DOI:** 10.1002/chem.202501322

**Published:** 2025-06-22

**Authors:** Aina Rebasa‐Vallverdu, Mattia Cattelan, Andrea Sartorel, Mauro Carraro, Marcella Bonchio, Stephen Mann, Pierangelo Gobbo

**Affiliations:** ^1^ School of Chemistry University of Bristol Bristol BS8 1TS UK; ^2^ Centre for Organized Matter Chemistry and Centre for Protolife Research School of Chemistry University of Bristol Bristol BS8 1TS UK; ^3^ Department of Chemical and Pharmaceutical Sciences University of Trieste and Interuniversity Consortium of Materials Science and Technology (INSTM) research unit of Trieste Via L. Giorgieri 1 Trieste 34127 Italy; ^4^ Department of Chemical Sciences University of Padova Via Marzolo 1 Padova 35131 Italy; ^5^ Interuniversity Consortium of Materials Science and Technology (INSTM) research unit of Padova and Institute of Membrane Technology ITM‐CNR UoS Padova Via Marzolo 1 Padova 35131 Italy; ^6^ Max Planck‐Bristol Centre for Minimal Biology School of Chemistry University of Bristol Cantock's Close Bristol BS8 1TS UK

**Keywords:** leaf‐like material, polyoxometalate coacervate vesicle, protocell, prototissue, water oxidation

## Abstract

The bottom‐up construction of leaf‐like materials capable of using visible light and water to produce oxygen and chemical energy is a major goal of artificial photosynthesis. In this work, we show that robust artificial cells (*protocells*) prepared by the membranization of polymer/nucleotide coacervate droplets with a bio‐inspired Ru‐based polyoxometalate (POM) catalytic oxygen‐evolving center are capable of continuous light‐assisted water oxidation at room temperature. We present a method to electrostatically assemble millions of the photocatalytic protocells into tissue‐like protocellular sheets and spheroids and demonstrate that the protocellular assemblies exhibit enhanced rates of photocatalytic water oxidation compared with individual protocells. Our results highlight opportunities for synergistically integrating key aspects of photocatalysis, cytomimetics, and bottom‐up engineering toward the development of modular photosynthetic active matter and applications in artificial photosynthesis and energy capture.

## Graphical abstract

Photocatalytic protocells (Ru_4_PCVs) incorporating a Ru‐based POM synzyme are constructed via the membranization of polymer/nucleotide coacervates. In the presence of a photosensitizer and a sacrificial electron acceptor, Ru_4_CVs are capable of continuously performing light‐induced water oxidation at room temperature. Assembled into tissue‐like sheets and spheroids, Ru_4_PCVs continue to perform light‐induced water oxidation, showing enhanced activity and higher‐order behaviours.

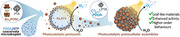

## 1 Introduction

The bottom‐up synthesis of bioinspired cell‐like entities (*protocells*) and their assembly into leaf‐like photosynthetic materials capable of converting sunlight into chemical fuel offer a cytomimetic approach that responds to the urgent issue of clean energy for a sustainable future.^[^ ^1^, ^2^ ^]^ Photosynthesis in plant cells relies on two fundamental protein centres: photosystem I, which is responsible for the photo‐reduction of nicotinamide adenine dinucleotide phosphate (NADP^+^), and photosystem II, which photo‐oxidizes water to dioxygen via a four‐electron mechanism (2H_2_O → O_2_ + 4H^+^ + 4e^‐^). The latter occurs at the oxygen‐evolving complex (PSII‐OEC), which consists of a distorted cubane Mn_4_CaO_5_ cluster that facilitates multiple cascade redox transformations with high efficiency and minimal energy cost.^[^ ^3^, ^4^, ^5^ ^]^ However, as photosystem II operates at an overpotential of 0.3–0.4 V, the dioxygen turnover progressively damages the protein, which therefore undergoes replacement every 30 minutes by the cell.^[^ ^6^, ^7^ ^]^

Given the chemical complexity of photocatalytic water oxidation and the intrinsic fragility of photosystem II, the design and bottom‐up construction of fully functioning photosynthetic protocells remains a major challenge. Recent studies have focused on the *ex‐situ* stabilization and integration of natural biological components such as photosystem II within synthetic microcompartments.^[^ ^8^, ^9^, ^10^, ^11^, ^12^ ^]^ For example, protocells capable of light‐induced adenosine triphosphate (ATP) production and carbon fixation were produced by coupling photosynthetic biological components (*e.g*., plant‐derived photosystem II, bacteriorhodopsin, or thylakoid membranes) to ATP synthase in protocell membranes.^[^ ^10^, ^12^ ^]^ While these pioneering works provided a route toward the first rudimentary photosynthetic protocells, the cytomimetic models displayed short lifetimes and low photocatalytic efficiencies due to the sacrificial degradation of photosystem II, irreversible build‐up of damaged photoactive proteins, and the instability of lipid vesicle scaffolds. In contrast, it should be possible to develop leaf‐like materials integrating long‐lasting, efficient photosynthetic protocells by implementation of a fully synthetic approach combining photosensitizers with stable bio‐inspired inorganic synthetic enzymes (synzymes) and their operation within robust protocell models.^[^ ^13^, ^14^, ^15^, ^16^ ^]^

**Scheme 1:**
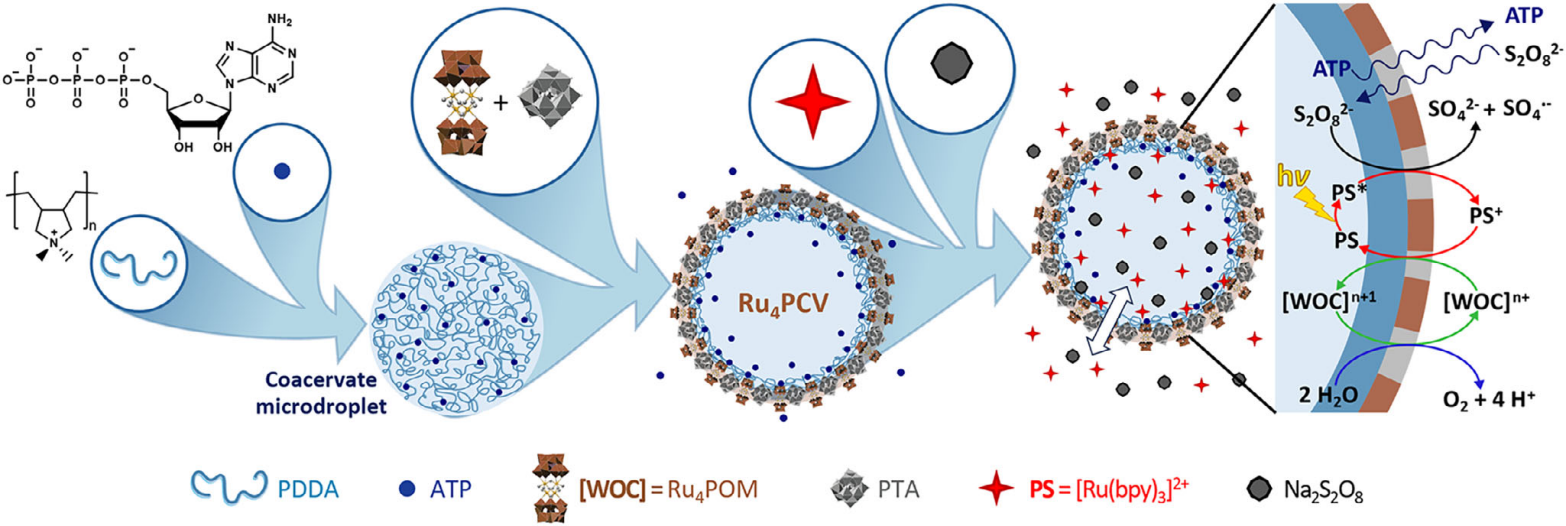
Assembly and photocatalytic activity of photocatalytic protocells. Scheme showing the spontaneous construction of Ru_4_PCVs by electrostatically mediated membranization and hypotonic swelling of PDDA/ATP coacervate microdroplets. Mixing PDDA (500 µL, 10 mM, pH 6.5) and ATP (500 µL, 10 mM, pH 6.5) produces membrane‐free coacervate microdroplets, which individually reconfigure into membrane‐bounded Ru_4_PCVs after addition of Ru_4_POM and PTA polyanions (Ru_4_POM: 20 µL, 12 mM, pH 6.5; PTA: 80 µL, 22 mM, pH 6.5).^19^ When placed in a solution containing [Ru(bpy)_3_]^2+^ (photosensitizer, PS) and S_2_O_8_^2‐^ (sacrificial electron acceptor or chemical fuel), the protocells perform a sequential electron transfer mechanism for the light‐driven water oxidation mediated by Ru_4_POM ([WOC]). The catalytic cycle revolves around a tetraruthenium(IV)‐oxo‐core and a sequence of four redox stages, with each step involving light‐induced excitation of [Ru(bpy)_3_]^2+^, subsequent oxidation of [Ru(bpy)_3_]^2+^ to [Ru(bpy)_3_]^3+^ by S_2_O_8_^2‐^, and reduction of [Ru(bpy)_3_]^3+^ back to [Ru(bpy)_3_]^2+^ by one‐electron oxidation of the tetraruthenium(IV)‐oxo‐core. Four sequential oxidations of the tetraruthenium(IV)‐oxo‐core by sequential electron transfer to photo‐generated [Ru(bpy)_3_]^3+^ enables water oxidation to O_2_ and protons.^23^

Herein, we report the bottom‐up construction of membranized coacervate‐based protocells capable of efficient and prolonged photocatalytic water oxidation at room temperature. The protocells consist of a molecularly crowded polydiallydimethylammonium chloride (PDDA)/adenosine 5′‐triphosphate (ATP) coacervate matrix with a central water‐filled lumen and external semi‐permeable membrane. Assembly of the membrane and formation of the vacuole occur spontaneously on addition of a polyoxometalate (POM) such as sodium phosphotungstate (PTA) to a suspension of PDDA/ATP coacervate droplets.^[^ ^17^, ^18^ ^]^ Catalytic activity is then introduced into the membrane by using a mixture of POMs that includes, besides PTA, a tetraruthenate cluster (Na_10_[Ru_4_(μ–O)_4_(μ–OH)_2_(H_2_O)_4_(γ–SiW_10_O_36_)_2_]; Ru_4_POM) using our previously established protocol to form catalytic Ru_4_POM‐containign polyoxometalate coacervate vesicles (Ru_4_PCVs).^[^ ^19^ ^]^ Significantly, Ru_4_POM consists of an adamantane‐like Ru‐oxo core that comprises four redox‐active sites that mimic the activity of the natural oxygen‐evolving photosynthetic Mn_4_CaO_5_ cluster of photosystem II.^[^ ^20^, ^21^, ^22^, ^23^ ^]^ In this paper, we demonstrate that uptake of a classical photosensitizer (tris(2,2′‐bipyridine)ruthenium(II), [Ru(bpy)_3_]^2+^) and chemical fuel (persulfate, S_2_O_8_^2‐^, as sacrificial electron acceptor) from the bulk solution into the Ru_4_PCVs results in the continuous production of O_2_ and protons from water oxidation activated by white light irradiation (Scheme 1). Importantly, the photocatalytic protocells operate continuously for several hours, are structurally and functionally robust, are easy to handle, and can be reused even after long‐term storage (>12–24 months). Given the above properties, we demonstrate that the Ru_4_PCVs can be electrostatically assembled into free‐standing protocellular sheets or spheroids comprising millions of tightly packed interconnected protocells with a tissue‐like micro‐architecture (*prototissues*). The protocellular assemblies display enhanced photocatalytic water oxidation compared to individual protocells, indicating that changes in prototissue microarchitecture impact the collective photocatalytic activity.

Taken together, our work builds upon our previously established method for generating catalytic Ru_4_PCVs^[^ ^19^ ^]^ and employing Ru_4_POM for water oxidation,^[20^ ^]^ advancing this foundation to create the first fully synthetic protocells and prototissues capable of *continuous* light‐induced water oxidation. This approach uniquely integrates core principles of photocatalysis, cytomimetic design, and bottom‐up engineering, representing a critical step toward *photosynthetic active matter*. By bridging these disciplines, our system opens new pathways for innovation in artificial photosynthesis and beyond.

## 2 Results and Discussion

### 2.1 Protocell‐mediated Photocatalytic Water Oxidation

**Figure 1:**
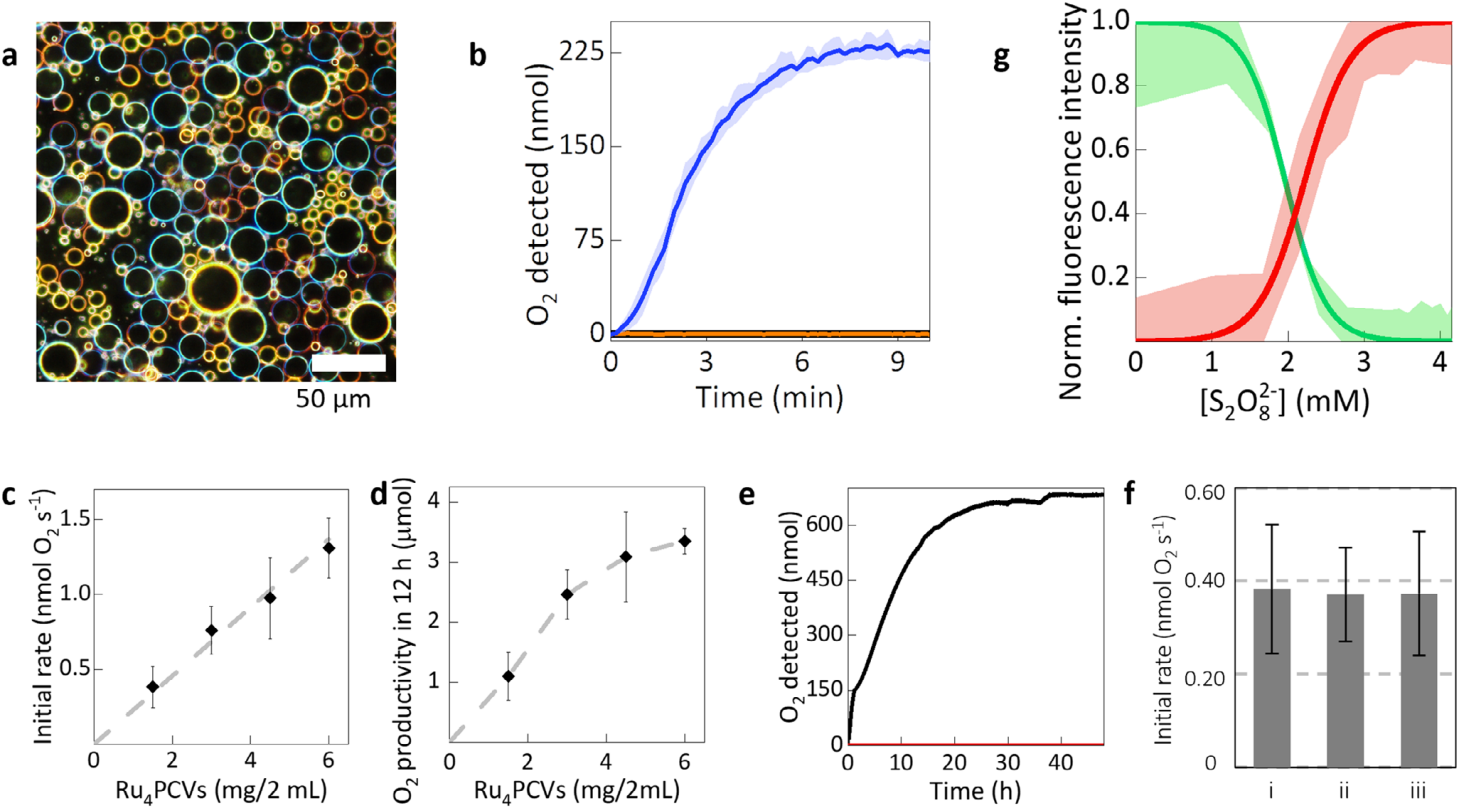
Protocell‐mediated photocatalytic water oxidation. a), Darkfield optical microscopy image showing a population of Ru_4_PCV in Milli‐Q water. b), Time‐dependent dioxygen production measured in‐solution for aqueous dispersions of Ru_4_PCVs (3 mg/2 mL, blue plot); Ru_4_PCVs (3 mg/2 mL) in the absence of light (control experiment, orange plot); and PTA‐CVs (3 mg/2 mL) in the absence of Ru_4_POM catalyst (control experiment, black plot). Error bands indicate standard deviation (n = 3 different experiments). All experiments were performed at room temperature in a sealed and dioxygen‐free photoreactor and in the presence of stirring. PCV dispersions were prepared in 1.85 mL of Na_2_SiF_6_/NaHCO_3_ buffer (3.8 mM in Na_2_SiF_6_ and 6.2 mM in NaHCO_3_, pH 5.6) containing 40 µL of a solution of Ru(bpy)_3_Cl_2_ (50 mM) and 100 µL of a solution of Na_2_S_2_O_8_ (100 mM), and stirred at 1,200 rpm. Samples in the photoreactor were irradiated with white light (2.78 mW cm^−2^ LED lamp). c), Plot of initial rates of dioxygen production against the amount of Ru_4_PCV used to prepare dispersions (volume = 2 mL). The initial rates were obtained from in‐solution measurements in the presence of stirring. Error bars indicate standard deviation (n = 3 different experiments). d), Graph of maximum yield of dioxygen produced against amount of Ru_4_PCVs used to prepare the dispersions (volume = 2 mL). The maximum amount of dioxygen produced was determined through headspace measurements in 12 hours, that is, with the reactor at equilibrium and in the presence of stirring. Error bars indicate standard deviation (n = 3 different experiments). e), Graph showing the amount of dioxygen produced in solution by a sample of 1.5 mg Ru_4_PCVs under diffusion‐controlled (no stirring) conditions over a 2‐day period of constant irradiation (black plot), compared to a control experiment performed under the same conditions but in the absence of PCVs (red plot). The control experiment highlights the excellent sealing of the reactor over such a long experiment period. f), Graph comparing the initial rate of dioxygen production obtained for batches of (i) fresh Ru_4_PCVs, (ii) Ru_4_PCVs after 2 y storage, and (iii) reused Ru_4_PCVs after an initial 7‐day photocatalytic experiment as shown in e). All experiments were carried out with the probe in solution, in the presence of stirring and 1.85 mL of Na_2_SiF_6_/NaHCO_3_ buffer (3.8 mM in Na_2_SiF_6_ and 6.2 mM in NaHCO_3_, pH 5.6) containing [Ru(bpy)_3_]^2+^ (final conc. 1 mM) and Na_2_S_2_O_8_ (final conc. 5 mM). Error bars indicate standard deviation (n = 3 different experiments). g), Plots showing changes in the normalized [Ru(bpy)_3_]^2+^ red fluorescence intensity for a population of [Ru(bpy)_3_]^2+^‐loaded Ru_4_PCVs (red plot) and normalized TNP‐tagged ATP green fluorescence intensity for a population of PTA‐CVs (green plot) as a function of S_2_O_8_^2‐^ concentration in bulk solution. The two experiments were conducted independently. The progressive increase in red fluorescence intensity arises from dissociation of quenched [Ru(bpy)_3_]^2+^/Ru_4_POM electrostatic pairs in the protocell membrane due to increases in ionic strength (S_2_O_8_^2‐^ concentration). Exchange of TNP‐tagged ATP (green fluorescence) by the uptake of S_2_O_8_^2‐^ results in expulsion of ATP and a loss of green fluorescence intensity in the coacervate matrix. Error bands indicate standard deviation over the PCV population, n = 100 PCVs.

Ru_4_POM‐containing protocells were prepared as previously reported.^[^ ^19^ ^]^ In brief, coacervate droplets prepared by mixing equimolar aqueous solutions of PDDA and ATP (10 mM) underwent transformation via the electrostatic complexation of an anionic PTA/Ru_4_POM mixture (7:1 molar ratio) with polycationic PDDA chains at the droplet surface. This process triggered changes in osmotic pressure, resulting in a complex three‐tiered microcompartment, comprising (1) a semi‐permeable, negatively charged Ru_4_POM/PTA/PDDA outer membrane, (2) a sub‐membrane PDDA/ATP coacervate shell, and (3) an internal aqueous lumen (Scheme 1). Brightfield and scanning electron microscopy (SEM) images confirmed that Ru_4_PCVs were intact, nonaggregated, polydisperse, hollow, birefringent spheres with a diameter of 25 ± 6.6 µm and a membrane thickness of 650 ± 150 nm (Figure 1a, Figure S1). Quantitative analysis indicated that 1 mg of the Ru_4_PCVs contained 8 nmol of Ru_4_POM with a PTA: Ru_4_POM molar ratio of 1.1 ± 0.04 (Figure S2). Typically, yields of 1.5 ± 0.5 mg of Ru_4_PCVs were obtained for each preparation.

Given the known catalytic properties of Ru_4_POM,^20^, ^21^, ^22^, ^23^, ^24^, ^25^, ^26^ we investigated whether integration of the synzyme into the coacervate vesicle membrane would provide a step toward synthetic protocells capable of efficient light‐assisted water oxidation. To test this, we irradiated aqueous suspensions of the protocells (3 mg) in the presence of [Ru(bpy)_3_]^2+^ (photosensitizer, final concentration 1 mM) and S_2_O_8_^2–^ (sacrificial electron acceptor, final concentration 5 mM), and used a probe to monitor oxygen production over time at room temperature both in the reaction medium (“in‐solution”) and headspace (Figure 1b, Figure S3). Typically, in the in‐solution experiments, dioxygen was produced at an initial rate of 0.76 ± 0.16 nmol s^−1^ with the oxygen concentration reaching a plateau value of 226 ± 7 nmol after 5 minutes (Figure 1b).

In contrast, no dioxygen was detected in the in‐solution control experiments carried out in the absence of light irradiation or by using PCVs prepared from PTA instead of a Ru_4_POM/PTA mixture (PTA‐CVs) (Figure 1b), confirming the competent oxygenic activity of Ru_4_POM within the protocell membrane. The corresponding turnover frequency (TOF) was 31.6 ± 4.0  × 10^−3^ s^−1^. This was 5 times lower than the TOF determined for Ru_4_POM (155 ± 27 × 10^−3^ s^−1^) when used as a homogeneous catalyst under the same experimental conditions.^[^ ^22^, ^24^ ^]^ The initial rate of dioxygen production increased linearly with the number of catalytic protocells present in the photoreactor (Figure 1c), whereas the TOFs remained constant, indicating that there were no competitive deactivation pathways inhibiting dioxygen production. The maximum amount of O_2_ released into the photoreactor headspace in 12 hours from 1.5 mg of Ru_4_PCVs was 1.1 ± 0.4 µmol, equivalent to a turnover number (TON) of 90 ± 9. The maximum amount of dioxygen produced in the headspace over 12 hours increased monotonically with the number of protocells present in the reactor until it reached a plateau value of 3.4 ± 0.7 µmol of O_2_ (Figure 1d). A similar plateau was observed under homogeneous catalysis conditions,^[^ ^21^ ^]^ which was associated with the limiting step of production of [Ru(bpy)_3_]^3+^. Significantly, control experiments performed by directly mixing all the components required for in situ protocell assembly (Ru_4_POM, PTA, PDDA, and ATP) in a photoreactor containing an aqueous solution of the photosensitizer and sacrificial electron produced negligible amounts of dioxygen (Figure S4). This was ascribed to the absence of protocell assembly under these conditions and the concomitant formation of irregular aggregates of the POM/polymer complex^[^ ^18^ ^]^ and highlighted the key role of the continuous PCV membrane in photocatalysis.

We verified that the photocatalytic activity was localized to the PCV system and not due to leakage of the Ru_4_POM catalyst into the bulk aqueous solution. For this, Ru_4_PCVs were filtered after performing a photocatalysis experiment, and the photocatalysis experiment was then repeated using the filtered solution. Significantly, no dioxygen was detected, confirming that Ru_4_POM remained bound within the protocell membrane (Figure S5). We ascribed this to the strong electrostatic interaction between Ru_4_POM and PDDA. This was further confirmed by incubating PTA‐CVs (colorless) in a solution of the more negatively charged Ru_4_POM (dark brown), which resulted in coloration of the protocells due to progressive uptake of the synzyme into the PTA/PDDA membrane (Figure S6). The resiliency and reusability of the Ru_4_PCVs (1.5 mg) were determined by monitoring the in‐solution photo‐assisted production of dioxygen over 2 days under standard photocatalysis conditions, but in the absence of stirring to avoid damaging the protocells (Figure 1e). Under these experimental conditions, we observed clear and continuous oxygen production for approximately 24 hours before the system reached a plateau, despite the known decomposition pathways of [Ru(bpy)_3_]^3+^ in the presence of light and persulfate.^[^ ^27^, ^28^ ^]^ The initial oxygen evolution rate of 0.033 ± 0.040 nmol s^−1^ was lower than the stirred system (Figure 1c), reflecting additional mass transport limitations in the static configuration. We attribute the system's sustained activity to two key factors: (1) the PCV membrane's compartmentalization effect, which spatially organizes the reactants and potentially mitigates decomposition pathways through selective absorption and regeneration of active species, and (2) the dynamic exchange of photosensitizer and persulfate with the bulk solution, which helps maintain catalytic turnover despite partial photosensitizer degradation. After two days, the solution became saturated with O_2_, reaching a value of 666 ± 9 nmol, which was higher than that detected in the absence of stirring (Figure 1b), which disfavored an effective exchange of the dioxygen produced between the reaction solution and the reactor headspace. The system was then left under irradiation for another 5 days, during which bubbles of oxygen formed on the inner walls of the photoreactor. The Ru_4_PCVs were then isolated, washed, and reused for a second photocatalytic experiment. Significantly, the activity of the Ru_4_PCVs remained unchanged, and no difference in the initial rate of O_2_ production was observed compared to the pristine protocells, highlighting the photostability and resilience of the protocells (Figure 1f). We also tested the catalytic activity of a sample of Ru_4_PCVs that were left in suspension in Milli‐Q water for > 12–24 months prior to use; no differences in light‐assisted water oxidation were observed when compared to a freshly prepared sample of the protocells (Figure 1f).

To elucidate the steps involved in protocell‐mediated water oxidation, we investigated the uptake capacity of the Ru_4_PCVs when exposed to the [Ru(bpy)_3_]^2+^ photosensitizer and S_2_O_8_^2‐^ sacrificial electron acceptor. Incubation of the catalytic protocells in aqueous [Ru(bpy)_3_]^2+^ for 2 hours produced Ru_4_PCVs with a homogeneous red fluorescent lumen and non‐fluorescent membrane (Figure S7a). In contrast, immersion of Ru_4_POM‐free PTA‐CVs for 2 hours in a solution of the photosensitizer produced a similar level of sequestration into the lumen along with the appearance of a clearly defined red fluorescent membrane (Figures S7b, and S8). The latter observation suggested that electrostatic interactions between Ru_4_POM and [Ru(bpy)_3_]^2+^ in the outer shell gave rise to fluorescence quenching specifically in the membrane of the photocatalytic protocells.^[^ ^23^ ^]^ This was confirmed by increasing the ionic strength (electrostatic screening) by subsequent addition of the Na_2_S_2_O_8_ sacrificial electron acceptor (< 4 mM), which gave rise to protocells with a red fluorescent membrane (Figures 1g, and S9) and minimal release of the photosensitizer into bulk solution. A progressive decrease in fluorescence intensity was observed at Na₂S₂O₈ concentrations above 4 mM, consistent with the known quenching effect of excess persulfate on [Ru(bpy)₃]^2^⁺ fluorescence.^[^ ^29^ ^]^ In contrast, the addition of Na₂S₂O₈ triggered the release of ATP from the coacervate matrix (Figure 1g). This observation confirms that persulfate uptake by [Ru(bpy)₃]^2^⁺‐loaded Ru_4_PCVs occurs through an anion exchange mechanism with ATP. This interpretation was supported by a control experiment where an aqueous 0.2 wt% TNP‐ATP solution showed only minimal fluorescence reduction upon persulfate addition (Figure S10), demonstrating that the significant ATP release observed in our PCV system cannot be attributed to direct persulfate‐ATP FRET interactions. Significantly, transfer of the [Ru(bpy)_3_]^2+^‐loaded PCVs into Milli‐Q water resulted in extremely slow release with *ca*. 50% of the photosensitizer remaining associated with the protocells after 24 days (Figure S11), indicating that sequestration of [Ru(bpy)_3_]^2+^ was irreversible under the experimental conditions employed.

Taken together, the above experiments indicated that the photocatalytic operation of the Ru_4_PCVs involved the uptake and co‐location of [Ru(bpy)_3_]^2+^ and chemical fuel (S_2_O_8_^2‐^) from the aqueous environment and concomitant activation of the Ru_4_POM/PDDA membrane and loss of ATP from the coacervate phase. Uptake of the electron acceptor also inhibited the formation of [Ru(bpy)_3_]^2+^/Ru_4_POM electrostatic pairs, favoring light‐assisted water oxidation at the protocell membrane. We also observed that direct irradiation of a suspension of Ru_4_PCVs (1.5 mg) preloaded with both [Ru(bpy)_3_]^2+^ and S_2_O_8_^2‐^ gave rise to dioxygen production (Figure S12), indicating that photo‐induced generation of [Ru(bpy)_3_]^3+^ could occur directly within the protocell membrane and that the coacervate subshell can concentrate chemical species useful for photocatalysis and act as a microscale reservoir. However, the amount of dioxygen produced under these conditions was much lower than that produced by Ru_4_PCVs dispersed in a solution of 1 mM of [Ru(bpy)_3_]^2+^ and 5 mM of Na_2_S_2_O_8_. This was attributed to the limited amount of photosensitizer and chemical fuel that could be stored within the protocell membrane and utilized for photocatalysis under these conditions.

### 2.2 Photocatalytic Water Oxidation in Protocellular Assemblies

**Figure 2:**
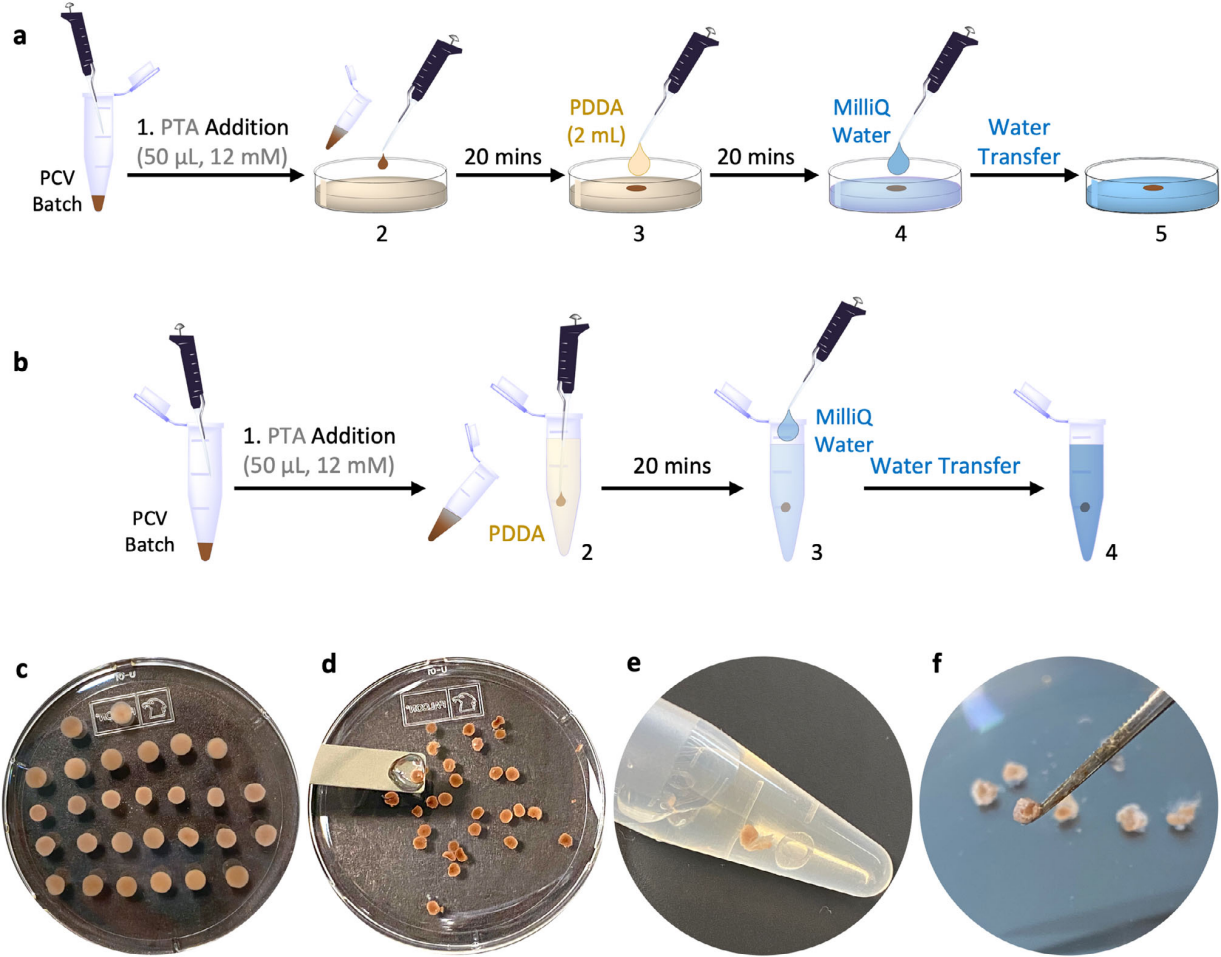
Higher‐order assembly of photocatalytic protocellular sheets and spheroids. a), Scheme showing the electrostatically induced assembly of Ru_4_PCVs into 2D protocellular sheets. Addition of aqueous PTA solution (50 µL, 12 mM, pH 6.5) to a freshly prepared suspension of Ru_4_PCVs (1.5 mg) in Milli‐Q water (14 µL) (**1**) followed by drop‐casting of the PCV/PTA suspension onto aqueous PDDA (2 mL, 450–500 kDa, 20 wt%) (**2**), incubation for 20 minutes, and addition of further PDDA produces a mechanically stable protocellular sheet (**3**). The sheet is left for a further 20 minutes, followed by the addition of Milli‐Q water (**4**). Aliquots of the resulting diluted solution are then removed, and the sheet picked up with a spatula and transferred to a Petri dish containing Milli‐Q water (**5**). b), Scheme showing the assembly of photocatalytic protocellular spheroids. The procedure is as shown in a) with the exception that the Ru_4_PCVs/PTA suspension is injected after immersion in aqueous PDDA (450‐500 kDa, 20 wt%). c,d), Photographs of a 35 mm Petri dish containing semi‐circular photocatalytic protocellular sheets dispersed in aqueous PDDA c), and after transfer into water d). e,f), Photographs of an Eppendorf tube containing photocatalytic protocellular spheroids in PDDA e) and free‐standing spheroids after transfer to water and removal with tweezers f).

Having established a bottom‐up method to fabricate discrete membrane‐bounded protocells capable of photocatalytic water oxidation, we explored the possibility of employing the Ru_4_PCVs as functional building blocks for the construction of protocellular assemblies with collective photocatalytic properties. For this, we used a four‐step process in which suspensions of the negatively charged Ru_4_PCVs (‐35.6 ± 1.4 mV),^[^ ^19^ ^]^ in the presence of PTA were drop‐casted onto or injected into aqueous solutions of polycationic PDDA to electrostatically assemble the individual protocells into free‐standing sheets or spheroids with sizes typically between 2–10 mm and 1–2 mm, respectively (Figure 2).

**Figure 3:**
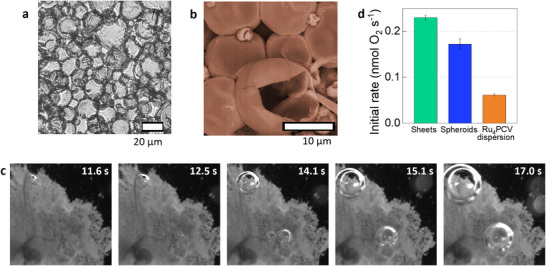
Characterization and photocatalytic activity of Ru_4_PCV‐based protocellular sheets. a,b), Brightfield microscopy a) and SEM b) images of the central section of a protocellular sheet showing intact Ru_4_PCVs; tight protocell‐protocell adhesions are observed throughout the sheet. c), Video frames showing the formation of dioxygen bubbles at the surface of a protocellular sheet after different time intervals of irradiation with white light (2.78 mW cm^−2^ LED lamp) in the presence of Ru(bpy)_3_Cl_2_ and Na_2_S_2_O_8_ (Video S1). d), Initial rates of dioxygen production for a dispersion of Ru_4_PCVs (1.5 mg /2 mL; orange plot), protocellular spheroids (blue plot), and protocellular sheets (green plot). Error bars indicate standard deviation (n = 3 different experiments). All the experiments were performed at room temperature in a sealed and dioxygen‐free photoreactor containing 1.85 mL of Na_2_SiF_6_/NaHCO_3_ buffer (3.8 mM Na_2_SiF_6_, 6.2 mM NaHCO_3_, pH 5.6) and a solution of Ru(bpy)_3_Cl_2_ (40 µL, 50 mM) and Na_2_S_2_O_8_ (100 µL, 100 mM). The photoreactor was irradiated with white light (2.78 mW cm^−2^ LED lamp). All experiments were performed in the absence of stirring to avoid damaging the materials.

In general, the sheets and spheroids could be stored for several months without loss of structural integrity, were resilient to low levels of mechanical stress, and could be readily handled with tweezers. Brightfield microscopy and SEM imaging (Figure 3a, b; Figures S13, S14), along with fluorescence microscopy images (Figure S15), showed that the protocellular sheets and spheroids consisted of millions of interconnected Ru_4_PCVs with tight protocell‐protocell membrane adhesions. In both cases, the Ru_4_PCVs remained structurally and morphologically intact and were organized in closely packed arrangements.

Given the structural integrity of the Ru_4_PCV‐based prototissues, we investigated whether the protocellular sheets could perform photo‐assisted water oxidation in the presence of [Ru(bpy)_3_]^2+^ and S_2_O_8_^2‐^ at room temperature. Exposure of the sheets to white light for several seconds produced oxygen bubbles across the surface of the films (Figure 3c, Video S1).

In contrast, the ability to effectively and rapidly nucleate dioxygen bubbles was not observed when the same experiment was performed with a dispersed population of Ru_4_PCVs, highlighting the emergent photocatalytic properties of the integrated leaf‐like material. Based on these observations, we sought to quantify the photocatalytic activity of the protocellular assemblies and investigate whether photocatalytic water oxidation was influenced by the macroscopic architecture of the prototissues. For this, we used 1.5 mg of Ru_4_PCVs to perform experiments using photocatalytic protocellular sheets, spheroids, or free Ru_4_PCVs. Time‐dependent curves of dioxygen production measured in solution indicated that the semi‐circular sheets performed best, followed by the spheroids, with the Ru_4_PCV dispersion showing the lowest photocatalytic activity (Figure 3d and Table 1). The initial rate, TOF, and TON of the protocellular sheets were higher than those of the protocellular spheroids. The improved performance of the sheets compared to the spheroids was attributed to the fact that the penetration of light and the diffusion of reagents to the core of the spheroids were more hindered. Moreover, the prototissue sheets and spheroids performed over three times better than free Ru_4_PCVs under the same reaction conditions. Taken together, these results provide evidence that the assembly of photocatalytic protocells into closely packed prototissues results in higher‐order behaviors, including enhanced water oxidation and the collective ability to rapidly nucleate dioxygen bubbles.

**Table 1:** Table summarizing the catalytic activity of Ru_4_POM, Ru_4_PCVs, protocellular sheets, and protocellular spheroids toward photoinduced water oxidation. All experiments were performed with the probe in solution, at room temperature, in a sealed and dioxygen‐free photoreactor, with a total of 12 nmol of Ru_4_POM catalyst, in 1.85 mL of Na_2_SiF_6_/NaHCO_3_ buffer (3.8 mM in Na_2_SiF_6_ and 6.2 mM in NaHCO_3_, pH 5.6) containing 40 µL of a solution of Ru(bpy)_3_Cl_2_ (50 mM) and 100 µL of a solution of Na_2_S_2_O_8_ (100 mM). Samples in the photoreactor were irradiated with white light (2.78 mW cm^−2^ LED lamp). Stirring was carried out at 1,200 rpm. Error indicates standard deviation (n = 3 different experiments).

System Investigated	Reactor Radius [cm]	Stirring	*Initial Rate* *[nmol O_2_ s^−1^]*	TOF [s^−1 ^× 10^−3^]
Ru_4_POM catalyst	0.5	Yes	1.55 ± 0.03	155 ± 27
1.5 mg Ru_4_PCVs	0.5	Yes	0.380 ± 0.100	31.6 ± 4.0
1.5 mg Ru_4_PCVs	0.5	No	0.033 ± 0.040	2.7 ± 0.3
1.5 mg Ru_4_PCVs	1.0	No	0.061 ± 0.002	5.1 ± 0.2
Protocellular sheets prepared from 1.5 mg Ru_4_PCVs	1.0	No	0.230 ± 0.001	19.0 ± 0.5
Protocellular spheroids prepared from 1.5 mg Ru_4_PCVs	1.0	No	0.170 ± 0.001	14.0 ± 1.0

## 3 Conclusions

In summary, we have significantly expanded upon our previous method for generating catalytic Ru_4_PCVs^[^ ^19^ ^]^ and using Ru_4_POM for water oxidation.^[20^ ^]^ We demonstrate that synzyme‐based photocatalytic protocells formed via the spontaneous membranization of Ru_4_POM coacervate droplets can sequester chemical fuel from their environment and, under light exposure, efficiently oxidize water at room temperature. Importantly, the Ru_4_PCVs are structurally and functionally resilient such that the protocells are capable of continuous oxygen production for 2 days, can be reused without loss of photo‐reactivity, and present a shelf life of over two years. These properties set a new benchmark in the area of photocatalytic protocells and demonstrate the potential of bioinspired synthetic approaches for the development of photosynthetic artificial cells with long‐term photocatalytic stability. Moreover, we describe a novel procedure for the assembly of Ru_4_PCVs into rudimentary leaf‐like materials in the form of robust, free‐standing, semi‐circular 2D sheets and 3D spheroids comprising millions of tightly packed interconnected protocells. Significantly, the protocellular assemblies exhibit superior photocatalytic capabilities compared to suspensions of individual protocells, such that the sheets and spheroids are capable of effective water oxidation and rapid release of dioxygen bubbles. Overall, our results indicate that the observed photocatalytic properties are modulated by changes in microarchitecture, possibly by influencing light absorption, sequestration of fuel molecules, reaction‐diffusion pathways, and removal of waste products. Moreover, in agreement with previous studies,^[^ ^30^, ^31^ ^]^ the chemical integration of individual protocells into tissue‐like materials with specific 3D architectures gives rise to higher‐order behaviors,^[^ ^32^ ^]^ suggesting that the implementation of collective interactions at the microscale will be an important consideration for the design of next‐generation leaf‐like biomimetic materials. From a general perspective, the cytomimetic fabrication of discrete photosynthetic protocells and prototissues from synthetic and resilient molecular components opens up opportunities for synergistically combining key aspects of photocatalysis, soft matter chemistry, and bottom‐up bioengineering. Our work also provides a step to photosynthetic active matter^[^ ^33^ ^]^ and paves the way to new developments in energy science and microreactor technology with potential applications in hydrogen production and carbon capture.

## 4 Methods

### Preparation of Ru_4_POM‐containing Polyoxometalate Coacervate Vesicles (Ru_4_PCVs)

A Ru_4_POM/PTA aqueous solution was freshly prepared by mixing 20 µL of a Ru_4_POM stock solution (12 mM, pH 6.5) and 80 µL of PTA stock solution (22 mM, pH 6.5) in Milli‐Q water. In a vial, 500 µL of a PDDA solution (Milli‐Q water, 10 mM, pH 6.5) and 500 µL of an ATP solution (Milli‐Q water, 10 mM, pH 6.5) were mixed and stirred for 30 s at 1,700 rpm to produce a turbid suspension of membraneless coacervate microdroplets. Subsequently, the Ru_4_POM/PTA aqueous solution was quickly injected into the vial, resulting in the instantaneous generation of membranized Ru_4_PCVs. The Ru_4_PCV dispersion was stirred for 30 s and then transferred into an Eppendorf tube and left to sediment for 30 minutes. The Ru_4_PCVs were then washed by removing the clear supernatant and redispersion in 850 µL of Milli‐Q water. The washing procedure was repeated three times to give a batch of Ru_4_PCVs dispersed in 500 µL of Milli‐Q water. Each batch of Ru_4_PCVs contained 1.5 ± 0.5 mg of PCVs as determined by lyophilizing and weighing the samples prepared as described above.

Samples prepared for confocal microscopy experiments and photocatalytic water oxidation experiments were prepared using the same procedure, with the exception that the Ru_4_PCVs were washed with PBS buffer (10 mM, pH 6.5) or Na_2_SiF_6_/NaHCO_3_ buffer (3.8 mM in Na_2_SiF_6_ and 6.2 mM in NaHCO_3_, pH 5.6), respectively, instead of Milli‐Q water.

### Electrostatic Assembly of Ru_4_PCVs Into Photocatalytic Protocellular Sheets or Spheroids

1.5 mg of Ru_4_PCVs were washed with Milli‐Q water, centrifuged (30 s, 1200 rpm) and the excess Milli‐Q water was removed. Subsequently, 50 µL of a solution of PTA (Milli‐Q water, 12 mM, pH 6.5) were added to the Ru_4_PCVs, and the dispersion was gently mixed with a pipette.

To obtain a circular sheet of interconnected Ru_4_PCVs, 2 µL of the Ru_4_PCV dispersion were carefully drop‐casted onto 2 mL of PDDA solution (450‐500 kDa, 20 wt% in water), which was contained in a polystyrene Petri dish 35 mm in diameter. The Ru_4_PCVs were incubated in PDDA for 20 minutes and subsequently completely covered with an additional 2 mL of PDDA solution and then incubated for a further 20 minutes. The resulting semi‐circular sheets were transferred into Milli‐Q water by progressively diluting the PDDA with 1 mL of water and then removing 1 mL of the resulting diluted solution from the Petri dish. This process was repeated 10 times. After each dilution and before solvent removal, the Petri dish was gently shaken for 10 minutes to homogenize the solution. Finally, the protocellular sheets were gently picked up with a spatula and placed in a clean Petri dish containing 2 mL of Milli‐Q water.

To obtain spheroids of the interconnected Ru_4_PCVs, 10 µL of the Ru_4_PCV dispersion were injected into an Eppendorf tube containing 600 µL of PDDA solution (450‐500 kDa, 20 wt% in water) and then incubated for 20 minutes. The spheroids were then transferred to Milli‐Q water by progressively diluting the PDDA solution with 500 µL of water and then removing 500 µL of the resulting diluted solution from the Eppendorf tube. This process was repeated 10 times. Finally, the spheroids were individually gently picked up with a spatula and placed in a clean Eppendorf tube containing 1 mL of Milli‐Q water.

### Photocatalytic Water Oxidation Experiments

40 µL of a solution of Ru(bpy)_3_Cl_2_ (Milli‐Q water, 50 mM) and the required amount of Ru_4_PCVs were suspended in 1.85 mL of Na_2_SiF_6_/NaHCO_3_ buffer (3.8 mM in Na_2_SiF_6_ and 6.2 mM in NaHCO_3_, pH 5.6) and stirred at 1200 rpm in a reactor vessel (0.5 cm radius) fitted with a dioxygen FOSPOR‐R probe with a silicon overcoat. The suspension was kept in the dark and purged with nitrogen for 15 minutes. Subsequently, 100 µL of a pre‐purged solution of Na_2_S_2_O_8_ (Milli‐Q water, 100 mM) were added to the reactor, the reactor was sealed, and the dioxygen level was monitored for 5 minutes to acquire a baseline measurement. After 5 minutes of equilibration time, a white light (white LED lamp 442 µW, 2.78 mW cm^−2^) was turned on to trigger photocatalysis, and the dioxygen level was monitored for at least 1 hour (solution measurements) or 25 hours (headspace measurements) at room temperature. The same procedure was used to carry out photocatalytic water oxidation experiments using Ru_4_POM, with the exception that 10 µL of a freshly prepared Ru_4_POM stock solution (1.2 mM) in Milli‐Q water was used instead of the Ru_4_PCVs.

A similar procedure was used to carry out photocatalytic water oxidation experiments in the presence of protocellular sheets or spheroids, except that the reactor was purged by bubbling nitrogen for 30 minutes instead of 15 minutes, and the experiments were performed without stirring to avoid damaging the protocellular assemblies. All experiments were carried out using a wider reactor flask (1 cm radius) than used for the Ru_4_PCVs.

To directly compare the photocatalytic properties of the PCVs and protocellular assemblies, experiments were carried out for the PCVs, sheets, or spheroids in a 1 cm‐radius reaction flask under nonstirred conditions.

## Supporting Information

The authors have cited additional references within the Supporting Information.^[^ ^20^, ^34^, ^35^ ^]^

## Author Contributions

A.R‐V., M.Cat., A.S., M.Car. M.B., S.M., and P.G. conceived the experiments. A.R‐V. performed the experiments. A.R‐V., M.Cat., A.S., M.Car. M.B., S.M., and P.G. undertook data analysis, discussed the results, and contributed to drafts of the manuscript. A.R‐V., M.B., S.M., and P.G. wrote the final manuscript.

## Conflict of Interests

The authors declare no conflicts of interest.

## Supporting information



Supporting Information

Supporting Information

## Data Availability

The data that support the findings of this study are available from the corresponding author upon reasonable request.
