# Supplementary material for: Continuous Light‐Induced Water Oxidation in Polyoxometalate‐Based Photocatalytic Protocells and Prototissues
Source: Chemistry. 2025 Jun 22;31(39):e202501322. doi: 10.1002/chem.202501322 (PMC12258676; doi:10.1002/chem.202501322)
Supplement: Supplementary file 2 — Supporting Information [file CHEM-31-e202501322-s001.pdf]

Supplementary Information

Continuous light-induced water oxidation in polyoxometalate-based photocatalytic protocells and prototissues

Aina Rebasa-Vallverdu[1,2,3], Mattia Cattelan[1,4], Andrea Sartorel[4], Mauro Carraro[4,5], Marcella Bonchio[4,5]*, Stephen Mann[1,2,6]*, Pierangelo Gobbo[1,3]*

[1]*School of Chemistry, University of Bristol, Bristol, BS8 1TS (UK).*

[2]*Centre for Organized Matter Chemistry and Centre for Protolife Research, School of Chemistry, University of Bristol, Bristol BS8 1TS, UK.*

[3]*Department of Chemical and Pharmaceutical Sciences, University of Trieste, and Interuniversity Consortium of Materials Science and Technology (INSTM), research unit of Trieste, Via L. Giorgieri 1, 34127 Trieste, Italy.*

[4] *Department of Chemical Sciences, University of Padova, Via Marzolo 1, 35131 Padova, Italy*

[5] *Interuniversity Consortium of Materials Science and Technology (INSTM) research unit of Padova, and Institute of Membrane Technology, ITM-CNR UoS Padova, Via Marzolo 1, 35131 Padova, Italy.*

[6]*Max Planck-Bristol Centre for Minimal Biology, School of Chemistry, University of Bristol, Cantock's Close, Bristol BS8 1TS, UK.*

E-mails: pierangelo.gobbo@units.it; s.mann@bristol.ac.uk; marcella.bonchio@unipd.it.

Table of Contents

1. Supplementary methods *3*

S1.1 Materials and methods ... 3

S1.2 Inductively coupled plasma atomic emission spectroscopy (ICP-AES) data and analysis ... 4

S1.3 Preparation of sodium phosphotungstate (PTA) coacervate vesicles (PTA-CVs) ... 5

S1.4 Polyoxometalate exchange in PCVs ... 5

S1.5 Calculation of initial rate of dioxygen production, turnover frequency (TOF), maximum dioxygen produced, and turnover number (TON) ... 5

S1.6 Photocatalysis experiments on PDDA/ATP/POM aggregates ... 6

S1.7 Sequestration of $[Ru(bpy)_3]^{2+}$ photosensitizer by PTA-CVs ... 6

S1.8 Preloading of $[Ru(bpy)_3]^{2+}$ photosensitizer within the Ru_4PCV membrane ... 6

S1.9 Interaction of $Na_2S_2O_8$ with $[Ru(bpy)_3]^{2+}$-loaded PTA-CVs ... 6

S1.10 Generation of TNP-ATP tagged PTA-CVs ... 7

S1.11 Generation of Ru_4PCVs loaded with $[Ru(bpy)_3]^{2+}$ and $S_2O_8^{2-}$ ... 7

2. Supplementary Figures *8*

3. Supplementary Video *17*

Video S1: Photocatalytic production of dioxygen bubbles by a 2D photocatalytic protocellular sheet ... 17

4. References *18*

1. Supplementary methods

S1.1 Materials and methods

The following materials were purchased from commercial sources and used as received unless otherwise stated. Sodium phosphotungstate hydrate (≥ 99.9%) (PTA), adenosine 5'-triphosphate disodium salt hydrate (99 %) (ATP), poly(diallyldimethylammonium chloride solution (average M_w 100 – 200 kDa 20 wt% in H_2O) (PDDA), poly(diallyldimethylammonium chloride solution (average M_w 450 – 500 kDa, 20 wt% in H_2O) (PDDA), sodium persulfate ($Na_2S_2O_8$), sulphuric acid (98%) (H_2SO_4), sodium phosphate monobasic, sodium phosphate dibasic, and tungsten standard solution (1000 mg L^{-1}, in 5% HNO_3 and 2% HF) were purchased from Sigma Aldrich (Merck). 2',3'-O-(2,4,6-trinitrophenyl)adenosine-5'-triphosphate tetra(triethylammonium) salt (TNP-ATP) was purchased from Bio-Techne. Sodium hexafluorosilicate (99%) (Na_2SiF_6), sodium carbonate (Na_2CO_3), and sodium hydrogen carbonate ($NaHCO_3$) were purchased from Alfa Aesar. Ruthenium standard solution (1,000 mg L^{-1}, in 5% HCl) for ICP-AES analysis was purchased from VWR International. Nitric acid (68%) (HNO_3) was purchased from Fischer Scientific. Tris(2,2'-bipyridine) dichlororuthenium (II) hexahydrate (98%) ([$(Ru(bpy)_3]Cl_2$) was purchased from Fischer Scientific and recrystallised from acetonitrile before being used. $Na_{10}[\{Ru_4O_4(OH)_2(H_2O)_4\}(\gamma\text{-}SiW_{10}O_{36})_2]$ (Ru_4POM) was synthesised according to a published procedure.[1]

Optical microscopy was performed with an Olympus BX35 microscope to obtain brightfield and phase contrast images at 5x, 10x, 20x or 40x magnification. Birefringence images were obtained using cross polarizing filters.

Widefield fluorescence microscopy was performed on a Leica DMI3000B Fluorescence Optical Microscope with a LEICA DFC7000 T-lamp equipped with 5x, 10x, 20x, and 40x lenses.

Fluorescence confocal microscopy was performed using a Leica SPE single channel confocal laser scanning microscope attached to a Leica DMi8 inverted epifluorescence microscope, equipped with solid state lasers 405 nm (25 mW) at 5x, 10x, 20x 40x or 63x magnification. The 40x and 63x lenses required the use of immersion oil (Type F, refractive index of 1.5180, purchased from Leica Microsystems). Time-dependent fluorescence images of $[Ru(bpy)_3]^{2+}$ were acquired using an excitation wavelength of 405 nm and an emission wavelength of 500 – 700 nm. Time-dependent fluorescence images of TNP-ATP fluorescence were acquired using an excitation wavelength of 405 nm and an emission wavelength of 500 – 600 nm.

All microscopy images were analysed with the latest version of ImageJ software.

ICP-AES analysis was performed on an Agilent 710 ICP-OES fitted with an Agilent SPS 3 auto-sampler. The instrument was calibrated using standard solutions of 0.3 ppm, 0.6 ppm, 0.9 ppm, 1.2 ppm and 1.5 ppm prepared from a single-element ruthenium standard solution (1,000 mg/L Ru in 5% HCl); and 20 ppm, 40 ppm, 60 ppm, 80 ppm and 100 ppm prepared from a single-element tungsten standard solution (1,000 mg/L W in 5% HNO_3 and 2% HF). Samples were prepared by digesting in H_2SO_4 (98%, 8 mL) lyophilized samples of PTA-CVs (9.7 mg) and Ru_4PCVs (9.9 mg) for 7 days. The resulting solutions were evaporated to almost dryness, re-dissolved in HNO_3 (1%, 25 mL) and filtered using hardened filter paper (Grade 54).

X-ray photoelectron spectroscopy was carried out using an Argus spectrometer working at a base pressure of 2.0×10^{-11} mbar. Core-level photoemission spectra were acquired in grazing incidence, *i.e.* 45° between the sample surface and the normal of the electron analyser, with a monochromatic Al Kα (1486.7 eV). The pass energy was set to 50, 20 and 100 eV for survey, high-resolution and high-throughput regions, respectively. The measurements were acquired at room temperature. The electron charging was neutralised by a flood gun. The binding energy scale was referenced to the carbon C-C bond in the C *1s* photoemission line at 284.8 eV.

Molecular oxygen concentration was measured using an OceanOptics NeoFox and FOSPOR-R probe with a silicon overcoat. In-solution measurements were carried out by calibrating the sensor using a two-point calibration before the measurements: the maximum dioxygen point (280 µmol L^{-1}) was acquired by reading the dioxygen level of the Na_2SiF_6-$NaHCO_3$ aqueous buffer solution; the minimum dioxygen point (0 µmol L^{-1}) was acquired after purging the aqueous buffer solution with nitrogen for 15 min. Headspace measurements were carried out by calibrating the sensor using a two-point calibration before the measurements: the maximum dioxygen point (20.9%) was acquired by reading the dioxygen level of the air; 0% dioxygen was

acquired after purging the reactor (solution and headspace) with nitrogen for 15 min. Data was logged using NeoFox Viewer version 2.40 averaging over 10 readings.

Samples for SEM were coated with 15 nm high purity graphite (Q150TES from Quorum Technologies ltd, UK) for backscattered electron analysis and to prevent charging. The SEM micrographs were taken on a JSM-IT300 (JEOL, Japan), operated at 15 kV, at a working distance of 10 mm, using both secondary and backscattered electron detectors.

S1.2 Inductively coupled plasma atomic emission spectroscopy (ICP-AES) data and analysis

Table S1: ICP-AES raw data for Ru_4PCVs.

Sample	Element	Concentration (ppm)	Intensity	Standard Deviation
Ru_4PCVs	Ru 240.272	1.46786	9,701.25	0.017796
Ru_4PCVs	Ru 245.657	0.866664	7,026.31	0.010138
Ru_4PCVs	Ru 267.876	1.56325	14,582	0.019805
Ru_4PCVs	W 207.912	18.7393	20,819.3	0.440985
Ru_4PCVs	W 220.449	18.3851	16,772.2	0.430216
Ru_4PCVs	W 224.876	18.15	4,679.75	0.356659
Blank	Ru 240.272	-0.010534	8.56606	0.000054
Blank	Ru 245.657	0.00225	16.5167	0.000205
Blank	Ru 267.876	-0.004312	14.8475	0.000044
Blank	W 207.912	-0.350604	66.2205	0.029017
Blank	W 220.449	-0.124404	57.0808	0.019012
Blank	W 224.876	0.699174	16.0919	0.011193

The raw data (see Table S1) were obtained as parts per billion (ppb) of ruthenium and tungsten. These values were converted to milligrams of ruthenium and tungsten, and subsequently to the corresponding molar values.

To obtain the amount of Ru_4POM per milligram of Ru_4PCV, first the moles of ruthenium were converted to moles of Ru_4POM synzyme, given a Ru:Ru_4POM ratio of 4:1. The resulting moles of Ru_4POM were then converted to milligrams using the Ru_4POM molecular weight of 5,690 g mol^{-1}, and then divided by the milligrams of Ru_4PCVs used in the experiment.

The amount of PTA in a sample of Ru_4PCVs was calculated using the same method starting from the moles of tungsten and the molecular weight of sodium phosphotungstate (2,880.2 g mol^{-1}).

The results of these calculations are summarized in Table S2 below.

Table S2: ICP-AES final results.

	Value	Error	
In 1 mg of Ru_4PCV there are:	4.60×10^{-2}	$\pm 9 \times 10^{-4}$	mg Ru_4POM
	8.09×10^{-9}	$\pm 2 \times 10^{-10}$	mol Ru_4POM
	4.6		% wRu_4POM/wPCVs
	2.61×10^{-2}	$\pm 7 \times 10^{-4}$	mg PTA
	9.08×10^{-9}	$\pm 3 \times 10^{-10}$	mol PTA
	2.6		% wPTA/wPCVs
Ratio W:Ru (mol)	8.4	± 0.8	

S1.3 Preparation of sodium phosphotungstate (PTA) coacervate vesicles (PTA-CVs)

PTA-CVs were produced using the procedure outlined in the main text methods section, with the exception that a freshly prepared PTA solution (100 µL, 22 mM pH 6.5) was used instead of the Ru_4POM/PTA mixture. Each sample batch prepared using this procedure yielded 1.5 ± 5 mg of PTA-CVs as determined by lyophilizing and weighing the samples.

S1.4 Polyoxometalate exchange in PCVs

To determine the exchange of PTA with Ru_4POM, 6.0 mg of PTA-CVs were prepared following the procedure described in Section S1.3, washed 3 times with Milli-Q water, and resuspended in 1,000 µL of Milli-Q water. To each sample, 20 µL of a solution of Ru_4POM in Milli-Q water (12 mM, pH 6.5, final concentration 0.25 mM) were added and the four samples were incubated on a shaker at 3,000 rpm for 5, 10, 30 or 60 min. The samples were subsequently centrifuged (30 s, 1,200 rpm) and washed with Milli-Q water 3 times.

To determine the exchange of Ru_4POM with PTA, 6.0 mg of Ru_4PCVs were prepared following the procedure described in the main text Method section, washed 3 times with Milli-Q water, and resuspended in 1,000 µL of Milli-Q water. To each sample, 100 µL of a PTA solution in Milli-Q water (22 mM, pH 6.5, final concentration 2.0 mM) were added and the four samples were incubated on a shaker at 3,000 rpm for 0.5, 1, or 4 h. The samples were subsequently centrifuged (30 s, 1,200 rpm) and washed with Milli-Q water 3 times.

S1.5 Calculation of initial rate of dioxygen production, turnover frequency (TOF), maximum dioxygen produced, and turnover number (TON)

The initial rate of dioxygen production was obtained from the slope of the linear part of the dioxygen curve obtained from solution measurements.

The TOF was calculated from the initial rate using the following formula:

$$TOF = \frac{Initial\ rate\ (\mu mol\ s^{-1})}{\mu mol\ catalyst}$$ (Supplementary Equation S1)

The maximum moles of dioxygen produced was obtained from the plateau region of the dioxygen curve obtained from headspace measurements.

The TON was calculated from the maximum dioxygen produced using the formula below:

$$TON = \frac{max\ \mu mol\ of\ O_2}{\mu mol\ of\ catalyst}$$ (Supplementary Equation S2)

S1.6 Photocatalysis experiments on PDDA/ATP/POM aggregates

In a reactor vessel fitted with a dioxygen FOSPOR-R probe with a silicon overcoat and equipped with a stirrer bar, 0.61 mg of PDDA, 24 µL of a Ru_4POM solution in Milli-Q water (1 mM), 2.02 mg of ATP, 26.7 µL of a PTA solution in Milli-Q water (1 mM), and 40 µL of a solution of $Ru(bpy)_3Cl_2$ in Milli-Q water (50 mM) were mixed in 1.85 mL of $Na_2SiF_6/NaHCO_3$ buffer (3.8 mM in Na_2SiF_6 and 6.2 mM in $NaHCO_3$, pH 5.6) and stirred at 1,200 rpm. The suspension was kept in the dark and purged with nitrogen for 15 min. Subsequently, 100 µL of a pre-purged solution of $Na_2S_2O_8$ in Milli-Q water (100 mM) were added to the reactor. The reactor was sealed, and the dioxygen level was monitored for 5 min to acquire a baseline. After 5 min of equilibration time a white light (white LED lamp 442 µW, 2.78 mW cm^{-2}) was turned on to trigger photocatalysis, and the dioxygen level was monitored for 1 h in solution.

The amounts of PDDA, ATP, Ru_4POM, and PTA utilised in these experiments were equivalent to the amounts of the same compounds present in the reactor when 3 mg of Ru_4PCVs were used.

S1.7 Sequestration of $[Ru(bpy)_3]^{2+}$ photosensitizer by PTA-CVs

Eleven samples of PTA-CVs were prepared following the procedure described in Section S1.3. Before washing, the PCV dispersions were transferred into different Eppendorf tubes and left to sediment for 30 min. The supernatant was carefully removed and replaced with 500 µL of Milli-Q water. Subsequently, 10 µL of a solution of $Ru(bpy)_3Cl_2$ in Milli-Q water (50 mM, pH 6.5, final concentration 1 mM) was added to each PTA-CV sample. The Eppendorf tubes were then placed on a shaker at 3,000 rpm for different amounts of time ranging from 1 min to 24 h to uptake increasing amounts of $[Ru(bpy)_3]^{2+}$. Finally, all samples were washed by centrifuging the PTA-CVs at 800 rpm for 30 s and by replacing the supernatant with PBS buffer (10 mM, pH 6.5). This washing procedure was repeated 3 times. The samples were then analysed *via* confocal fluorescence microscopy.

S1.8 Preloading of $[Ru(bpy)_3]^{2+}$ photosensitizer within the Ru_4PCV membrane

A Ru_4POM/PTA aqueous solution was freshly prepared by mixing 20 µL of a Ru_4POM stock solution (12 mM, pH 6.5) and 80 µL of a PTA stock solution (22 mM, pH 6.5) in Milli-Q water. In a 1.75 mL vial, 500 µL of a solution of PDDA in Milli-Q water (10 mM, pH 6.5) and 10 µL of a solution of $Ru(bpy)_3Cl_2$ in Milli-Q water (50 mM, pH 6.5) were mixed. To this solution, 500 µL of a solution of ATP in Milli-Q water (10 mM, pH 6.5) were added to afford a turbid orange suspension of $[Ru(bpy)_3]^{2+}$-containing coacervate microdroplets, which was stirred for 30 s at 1,700 rpm. Subsequently, the freshly prepared Ru_4POM/PTA aqueous solution was quickly injected into the vial, resulting in the instantaneous generation of a batch of Ru_4PCVs with $[Ru(bpy)_3]^{2+}$ encapsulated within the membrane. The sample was transferred to an Eppendorf tube placed on a shaker at 3,000 rpm for 24 h to uptake $[Ru(bpy)_3]^{2+}$. The sample was then washed by centrifuging the $[Ru(bpy)_3]^{2+}$-loaded Ru_4PCVs at 800 rpm for 30 s and by replacing the supernatant with PBS buffer (10 mM, pH 6.5). The washing procedure was repeated 3 times. The samples were then investigated by confocal fluorescence microscopy.

The same procedure was repeated to yield $[Ru(bpy)_3]^{2+}$-loaded PTA-CVs except that a freshly prepared PTA solution (100 µL, 22 mM pH 6.5) was used instead of the Ru_4POM/PTA mixture.

S1.9 Interaction of $Na_2S_2O_8$ with $[Ru(bpy)_3]^{2+}$-loaded PTA-CVs

20 µL of a suspension of $Ru(bpy)_3]^{2+}$-loaded PTA-CVs (soaking time = 1 h) prepared using the general procedure outlined in Section S1.8 were suspended in 500 µL of PBS (pH 6.5, 10 mM) and placed in a Petri dish and left to sediment for 10 minutes. Subsequently, 5 µL aliquots of a $Na_2S_2O_8$ solution in Milli-Q water (5 mM, pH 6.5) were added to the PCV dispersion until a total volume of 100 µL was reached. After each 5 µL addition the system was allowed to equilibrate for 1 min before confocal microscopy images were acquired (λ_{exc} = 405 nm, λ_{em} = 500 – 700 nm).

S1.10 Generation of TNP-ATP tagged PTA-CVs

Fluorescently TNP-ATP tagged PTA-CVs were produced using the general procedure outlined in Section S1.3, with the exception that a solution of ATP and TNP-tagged ATP in Milli-Q water (100 µL, pH 6.5, 22 mM overall, 0.2 mol% of TNP-tagged ATP) was used instead of only an ATP solution.

For confocal fluorescence microscopy experiments, 20 µL of a suspension of TNP-tagged ATP PTA-CVs in Milli-Q water were placed in a Petri dish for confocal microscopy imaging. The PTA-CVs were left to sediment for 10 min. Subsequently, 5 µL aliquots of a $Na_2S_2O_8$ solution in Milli-Q water (5 mM, pH 6.5) were added to the PCV dispersion until a total volume of 100 µL was reached. After each 5 µL addition the system was allowed to equilibrate for 1 min before microscopy images were acquired (λ_{exc} = 405 nm, λ_{em} = 500 – 600 nm).

S1.11 Generation of Ru_4PCVs loaded with $[Ru(bpy)_3]^{2+}$ and $S_2O_8^{2-}$

A batch of Ru_4PCVs preloaded with $[Ru(bpy)_3]^{2+}$ was prepared according to the method described in Section S1.8. Upon washing the Ru_4PCVs and in order to increase the amount of photosensitiser, the sample was dispersed in 1,000 µL of Milli-Q water and 10 µL of a solution of $[Ru(bpy)_3]Cl_2$ in Milli-Q water (110 mM, pH 6.5, final concentration 1 mM), and placed on a shaker at 3,000 rpm. After 24 h, the Ru_4PCVs were washed by removing the supernatant and redispersing them in 850 µL of Milli-Q water. The washing procedure was repeated 3 times. The $[Ru(bpy)_3]^{2+}$-loaded Ru_4PCVs were then dispersed in 600 µL of a $Na_2S_2O_8$ solution (16.7 mM in Milli-Q water) and placed on a shaker at 3,000 rpm for 24 h. The Ru_4PCVs were then washed by removing the supernatant and redispersing them in 850 µL of Milli-Q water to give a batch of $[Ru(bpy)_3]^{2+}$- and $S_2O_8^{2-}$-loaded Ru_4PCVs dispersed in 500 µL of Milli-Q water. Each batch of Ru_4PCVs prepared by this methodology contained 1.5 ± 0.5 mg of PCVs as determined by lyophilizing and weighing the samples prepared as described above.

2. Supplementary Figures

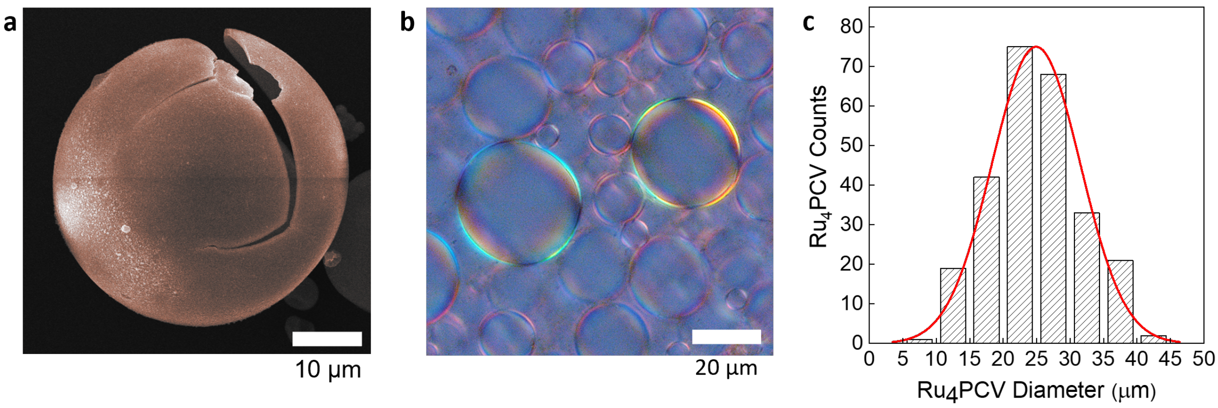

Figure S1. Characterisation of photocatalytic protocells. a, SEM image showing a lyophilized partially fractured Ru_4PCV with hollow interior and smooth outer membrane surface. The image has been artificially coloured to illustrate the natural tinge of the material. **b,** Representative polarised light microscopy image of Ru_4PCVs showing birefringence. **c**, Size distribution plot of a sample of Ru_4PCVs; mean diameter = 25 ± 6.6 µm.

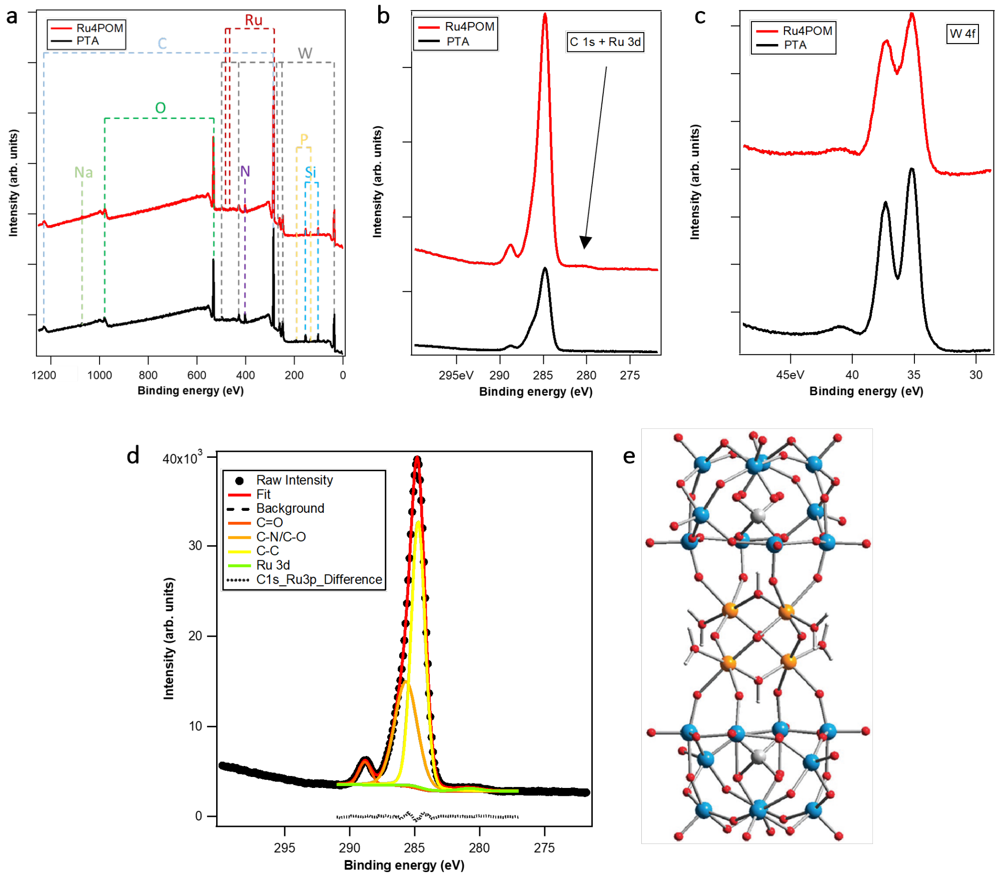

Figure S2. XPS characterisation of PCVs. **a**, XPS survey comparing the main photoemission lines detected for Ru_4PCVs (red spectrum) and PTA-CVs (black spectrum). The photoemission lines of the main elements (Ru, W, C, O, P, N, Si, Na) are labelled in the graph. Ru photoemission lines are almost not detectable by the survey, as they overlap with the intense C 1s signal. **b**, Comparison between the high-resolution scans of the C 1s region for Ru_4PCVs (red spectrum) and PTA-CVs (black spectrum). A shoulder peak at 281.3 eV in the Ru_4PCV spectrum is due to the Ru $3d_{5/2}$ photoemission line, which is absent from the corresponding spectrum of PTA-CVs.[2] **c**, Comparison between the high-resolution scans of the W 4f photoemission lines for Ru_4PCVs (red spectrum) and PTA-CVs (black spectrum), highlighting a prevalence of W(VI) in both samples (peak at 35.1 eV, W 4f).[3] **d**, Deconvolution of the C 1s and Ru 3d peaks for the Ru_4PCV sample. The deconvolution highlights the presence of carbon atoms belonging to different chemical environments: C–N and C–O from ATP and PDDA (orange curve), C–C from ATP and PDDA (yellow curve). Two typical peaks for the Ru 3d photoemission line at 285.5 eV (Ru $3d_{3/2}$) and 281.3 eV (Ru $3d_{5/2}$) are shown in green. The detected binding energies are typical of a Ru (IV) species,[2] indicating that the Ru_4POM did not decompose when complexed with the PDDA/ATP coacervate micro-droplet to form the PCV. **e**, 3D model of Ru_4POM. Yellow: ruthenium atoms; grey: silicon atoms; blue: tungsten atoms; red: oxygen atoms; white: hydrogen atoms (hydrogens are omitted from γ-SiW_{10} units). Analysis of the relative composition of W and Ru, after normalization of the individual photoemission lines by their sensitivity factor and inelastic mean-free path, gave a W/Ru atomic ratio of 11 ± 3, which agrees within experimental error with the ICP quantitative analysis. The high uncertainty on the measurement is due to low Ru signal. The large ratio is caused by the attenuation of the Ru signal exerted by the two γ-SiW_{10} caps.

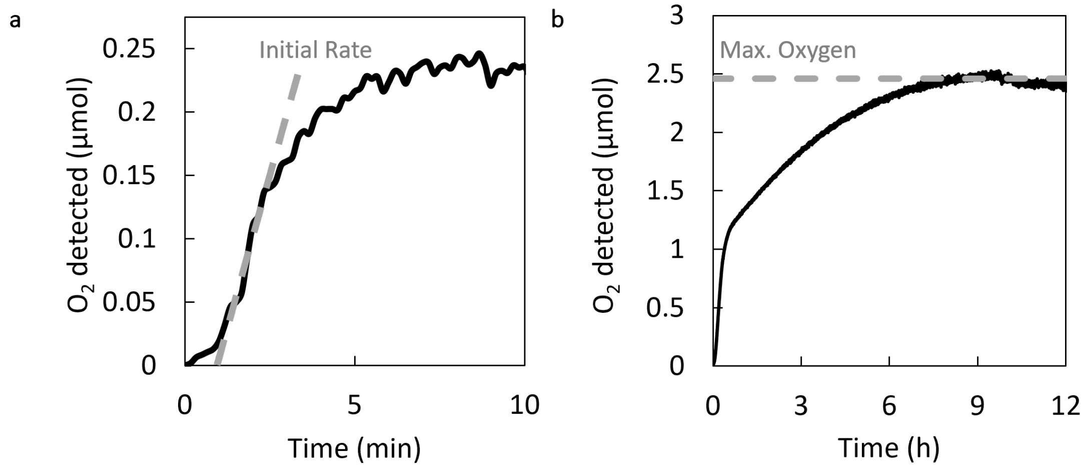

Figure S3. Calculation of initial rate of dioxygen production and maximum amount of oxygen produced. a, Representative plot of dioxygen produced over time by 3.0 mg of Ru_4PCVs dispersed in 1.85 mL of Na_2SiF_6/$NaHCO_3$ buffer (3.8 mM in Na_2SiF_6 and 6.2 mM in $NaHCO_3$, pH 5.6) containing 40 µL of a solution of $Ru(bpy)_3Cl_2$ (50 mM) and 100 µL of a solution of $Na_2S_2O_8$ (100 mM). For this measurement the dioxygen probe was inside the solution (solution measurement). The initial rate of dioxygen production was determined by the slope of the linear part of the curve as indicated by the dashed grey line. **b**, Representative plot of dioxygen produced over time for the system in (**a**). For this measurement the dioxygen probe was kept above the solution (headspace measurement). The maximum amount of dioxygen produced was determined by the plateau value of the curve as indicated by the dashed grey line.

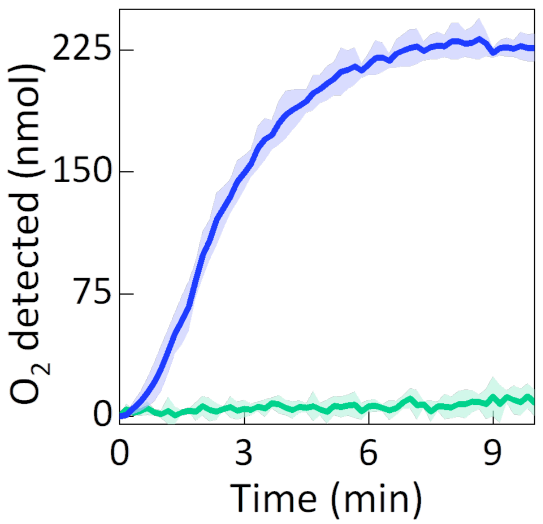

Figure S4. Photocatalysis control experiments. Graph comparing the amount of dioxygen produced in solution by a sample of Ru_4PCVs (3 mg, normal experiment, blue plot) and by a sample produced by mixing directly in the photoreactor 61 mg of PDDA, 2.02 mg of ATP, 24 µL of a Ru_4POM solution in Milli-Q water (1 mM, pH 6.5), and 26.7 µL of a PTA solution in Milli-Q water (1 mM, pH 6.5) (green plot). Error bands indicate standard deviation (n = 3 different experiments). All these photocatalysis experiments were carried out in the presence of 1.85 mL of Na_2SiF_6/$NaHCO_3$ buffer (3.8 mM in Na_2SiF_6 and 6.2 mM in $NaHCO_3$, pH 5.6) containing $[Ru(bpy)_3]^{2+}$ (final conc. 1 mM) and in $Na_2S_2O_8$ (final conc. 5 mM).

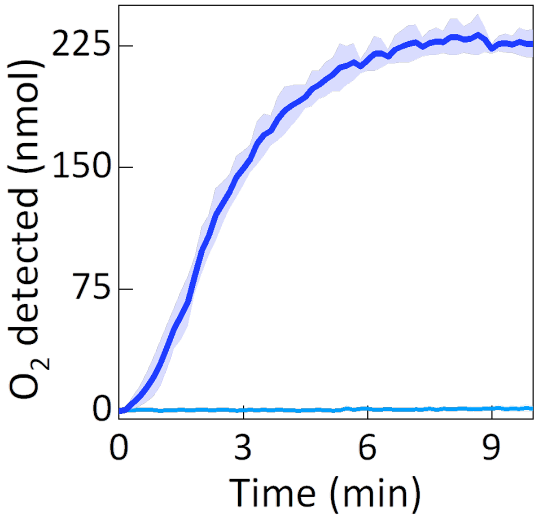

Figure S5. Photocatalysis control experiment. Graph comparing the amount of dioxygen produced in solution by a sample of Ru_4PCVs (3 mg, normal photocatalysis experiment, blue plot), and by the supernatant from a previously ran experiment (light blue). Error bands indicate standard deviation (n = 3 different experiments). All these photocatalysis experiments were carried out in the presence of 1.85 mL of $Na_2SiF_6/NaHCO_3$ buffer (3.8 mM in Na_2SiF_6 and 6.2 mM in $NaHCO_3$, pH 5.6) containing $[Ru(bpy)_3]^{2+}$ (final conc. 1 mM) and in $Na_2S_2O_8$ (final conc. 5 mM).

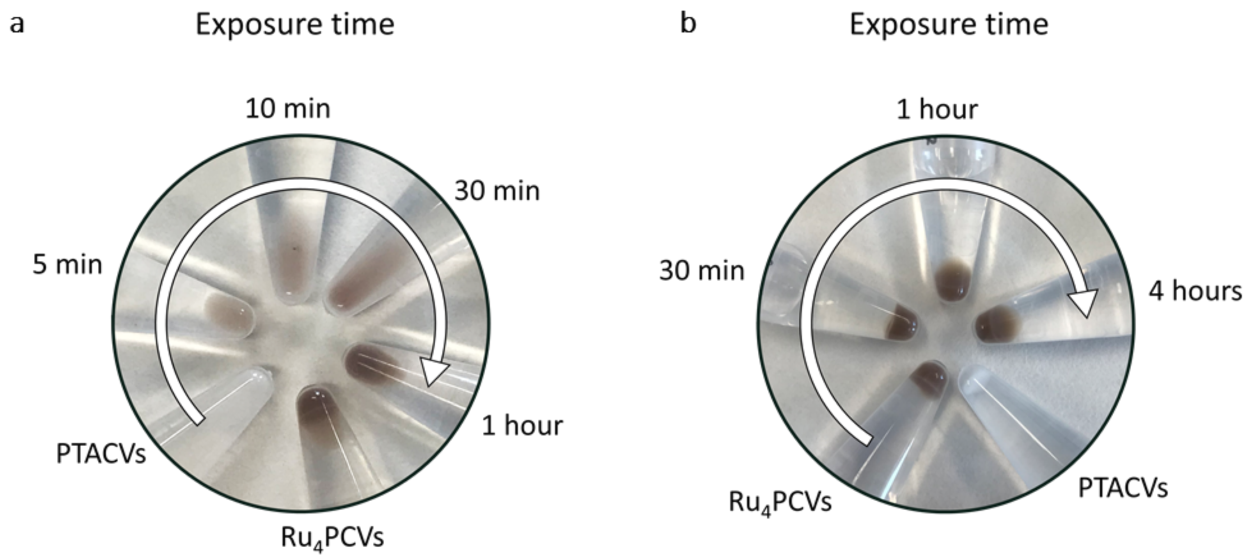

Figure S6. Polyoxometalate exchange experiments. a, Photograph showing circular array of Eppendorf tubes containing sedimented PTA-CVs in Milli-Q water after exposure to an aqueous solution of Ru_4POM (0.25 mM) for the times indicated in the figure. Longer exposure times transformed more of the white PTA-CVs into dark brown Ru_4PCVs. As-prepared samples of PTA-CVs and Ru_4PCVs are included in the picture for comparison. **b**, Photograph showing circular array of Eppendorf tubes containing sedimented Ru_4PCVs in Milli-Q water after exposure to an aqueous solution of PTA (2.0 mM) for the times indicated in the figure. In contrast with (**a**), no change in colour was observed despite the higher concentration of POM and the longer exposure times, indicating that Ru_4POM has a stronger binding constant to the PCV structure compared to PTA. As-prepared samples of PTA-CVs and Ru_4PCVs are included in the picture for comparison.

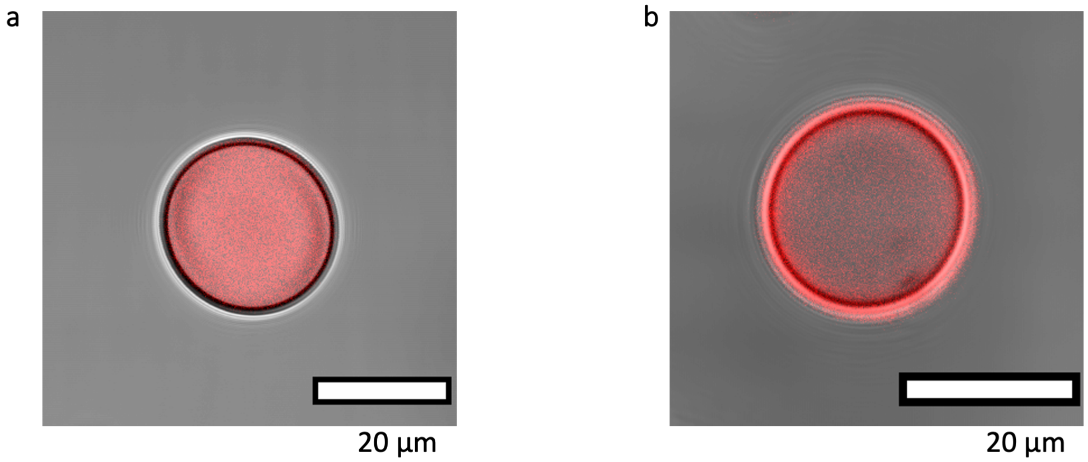

Figure S7. Photosensitizer uptake by coacervate vesicles. a, Confocal fluorescence microscopy image of a single Ru_4PCV after incubation for 2 h in a solution of $[Ru(bpy)_3]^{2+}$ (1 mM, red fluorescence; PBS buffer) showing a homogeneous distribution of the photosensitizer across the lumen of the protocell. Minimal fluorescence is observed at the Ru_4POM/PTA/PDDA membrane. **b,** Confocal fluorescence microscopy image of a single PTA-CV after incubation for 2 h in a solution of $[Ru(bpy)_3]^{2+}$ (1 mM, red fluorescence; PBS buffer) showing a homogeneous distribution of the photosensitizer across the protocell lumen and a higher concentration in the PTA/PDDA membrane.

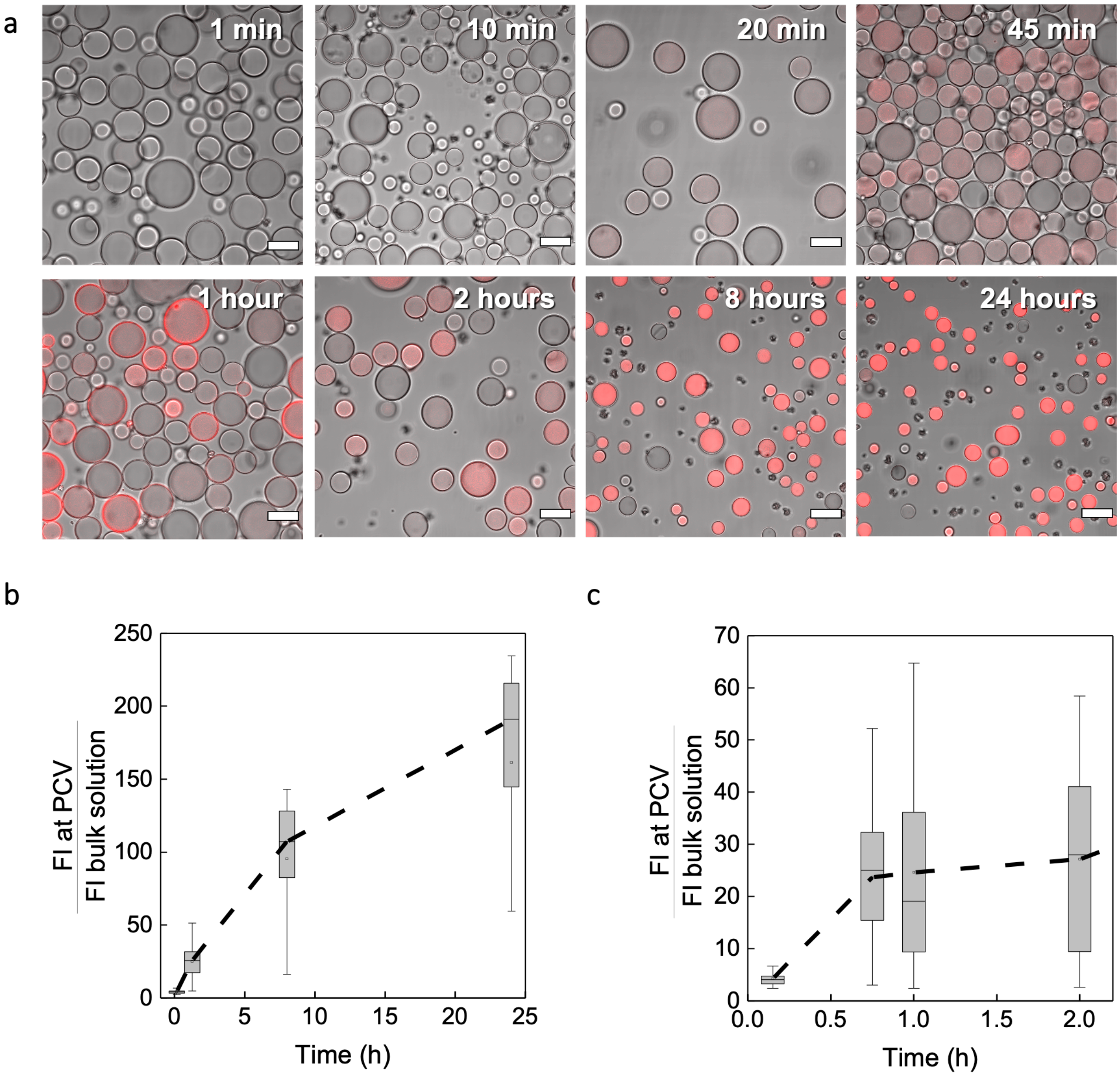

Figure S8. Kinetics of photosensitizer uptake by PTA-CVs. a, Time-dependent overlayed brightfield and fluorescence confocal images of a population of PTA-CVs after soaking in a solution 1 mM of $[Ru(bpy)_3]^{2+}$ for various times followed by washing with Milli-Q water prior to imaging. All scalebars are 20 µm. The images show a progressive uptake of the red fluorescent photosensitizer and sequestration inside and on the membrane of the PTA-CVs. **b**, Graph showing the time-dependent increase in $[Ru(bpy)_3]^{2+}$ fluorescence intensity (FI) ratio (FI = dye fluorescence intensity measured inside the PCV over dye fluorescence intensity measured in the bulk aqueous solution). A slow continuous uptake of $[Ru(bpy)_3]^{2+}$ (red fluorescence) into the PTA-CVs is observed with the red fluorescence intensity ratio (F_{in}/F_{out}) attaining typical values of 191 ± 64 after 24 h. All data points (n = 100-200 for each time point) in the 25% - 75% interquartile range are represented by the grey box. The median and mean are represented by a line and a square within the grey boxes, respectively. The minimum and maximum values are represented by the whiskers. **c**, Data from (**b**) plotted for time periods only up to 2 h. Statistical analysis is the same as in (**b**).

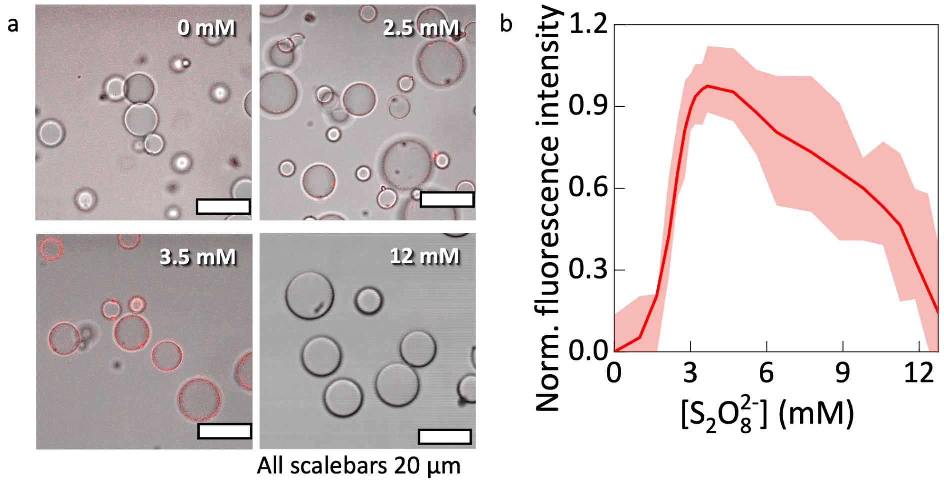

Figure S9 Interaction of $Na_2S_2O_8$ with Ru_4PCVs preloaded with $[Ru(bpy)_3]^{2+}$. a, Overlay of brightfield and confocal fluorescence images of $Ru(bpy)_3]^{2+}$-loaded Ru_4PCVs taken at different concentrations of $S_2O_8^{2-}$ present in the bulk solution as indicated on the top right corner of each image. **b,** Graph showing changes in the normalised $Ru(bpy)_3]^{2+}$ fluorescence intensity for a population of $Ru(bpy)_3]^{2+}$-loaded Ru_4PCVs as a function of the concentration of $S_2O_8^{2-}$ present in the bulk solution. The initial increase in the red fluorescence intensity of $Ru(bpy)_3]^{2+}$ is associated with the dissociation of the $[Ru(bpy)_3]^{2+}$/Ru_4POM electrostatic pairs at the PCV membrane. The subsequent loss of fluorescence is associated to the quenching of $[Ru(bpy)_3]^{2+}$ by persulfate ions. Error bands indicate standard deviation (n = 3 different experiments).

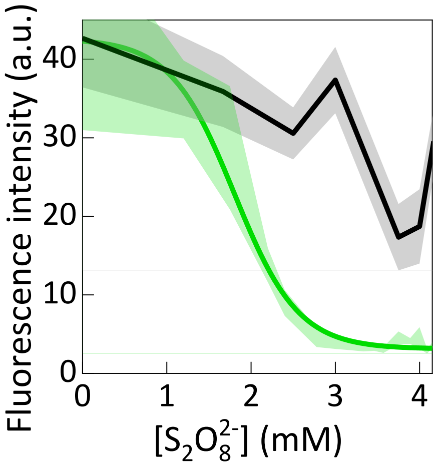

Figure S10 Interaction between $Na_2S_2O_8$ and TNP-ATP. Graph comparing the fluorescence intensity of a population of PTA-CVs containing TNP-tagged ATP (green plot) with a 0.2% TNP-ATP aqueous solution (black plot, control experiment) as a function of $S_2O_8^{2-}$ concentration present in the bulk aqueous solution. Error bands indicate standard deviation (n = 3 different experiments). The two experiments were conducted independently. The fluorescence intensity of the aqueous TNP-ATP solution shows only minimal reduction upon addition of persulfate and has minimal overlap with the PTA-CVs data, confirming that the fluorescence decrease observed in the PTA-CVs containing TNP-tagged ATP is primarily a result of ion exchange effects between negatively charged ATP and persulfate.

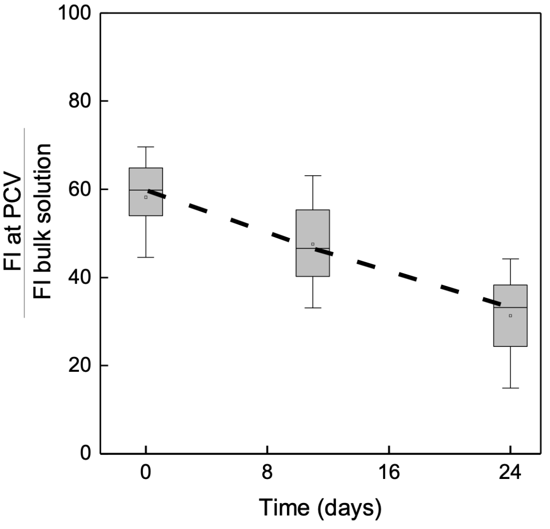

Figure S11. Kinetics of $[Ru(bpy)_3]^{2+}$ release from PCVs. Graph showing the trend of the time-dependent release of $[Ru(bpy)_3]^{2+}$ from $[Ru(bpy)_3]^{2+}$-loaded PTAPCVs. The $[Ru(bpy)_3]^{2+}$-loaded PTAPCVs were washed and left in Milli-Q water for up to 24 h. The change in $[Ru(bpy)_3]^{2+}$ fluorescence intensity (FI) ratio, calculated as the ratio between the fluorescence intensity of the dye inside the PCV over the fluorescence intensity of the dye in the bulk aqueous solution. All the data points (n = 100-200 for each time point) in the 25% - 75% interquartile range are represented by the grey box. The median and mean are represented by a line and a square within the grey boxes, respectively. The minimum and maximum are represented by the whiskers.

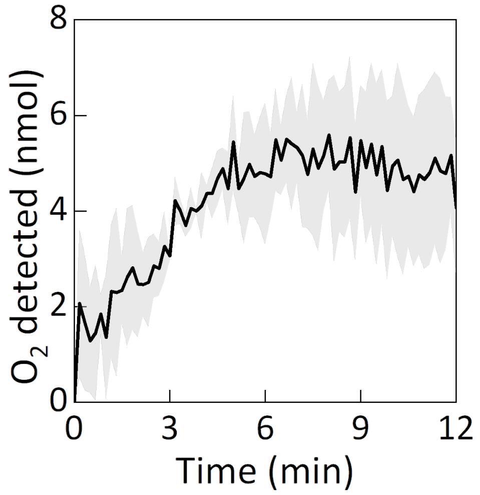

Figure S12. Photocatalysis using a population of Ru_4PCVs preloaded with chemical fuel. Plot showing the amount of dioxygen produced over time by a population Ru_4PCVs (1.5 mg) preloaded with both $[Ru(bpy)_3]^{2+}$ nd $Na_2S_2O_4$, dispersed in 1.85 mL of $Na_2SiF_6/NaHCO_3$ buffer (3.8 mM in Na_2SiF_6 and 6.2 mM in $NaHCO_3$, pH 5.6) and irradiated with white light (2.78 mW cm^{-2}). Error bands indicate standard deviation (n = 3 different experiments).

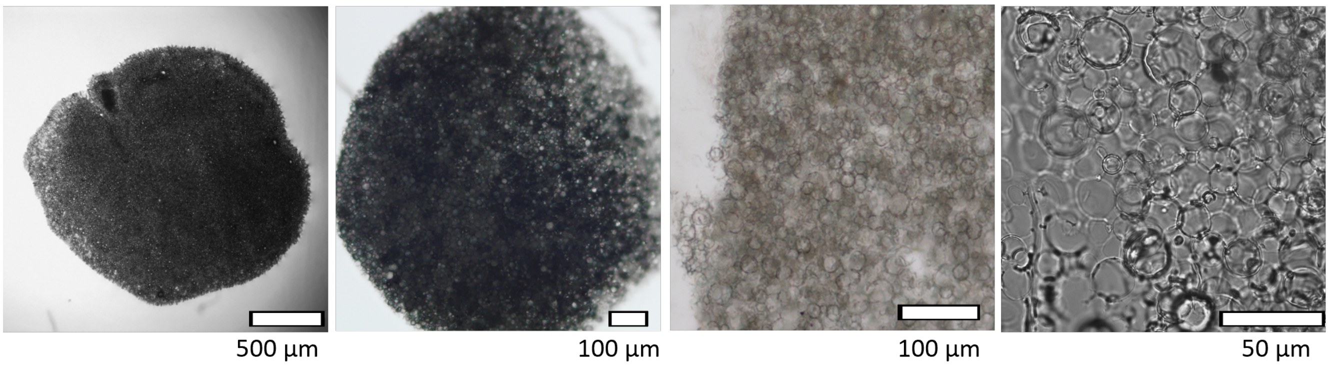

Figure S13. Photocatalytic protocellular sheets. Brightfield microscopy images recorded at different magnifications showing self-standing extended assemblies of interconnected Ru_4PCVs.

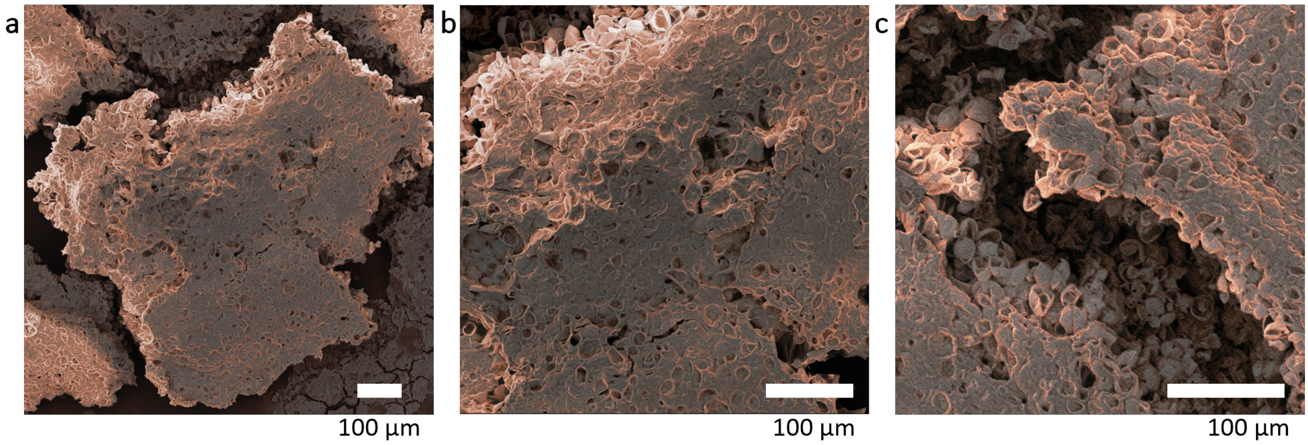

Figure S14. Photocatalytic protocellular sheets. SEM images of air-dried prototissue sheets showing the dense interlinked array of collapsed Ru_4PCVs.

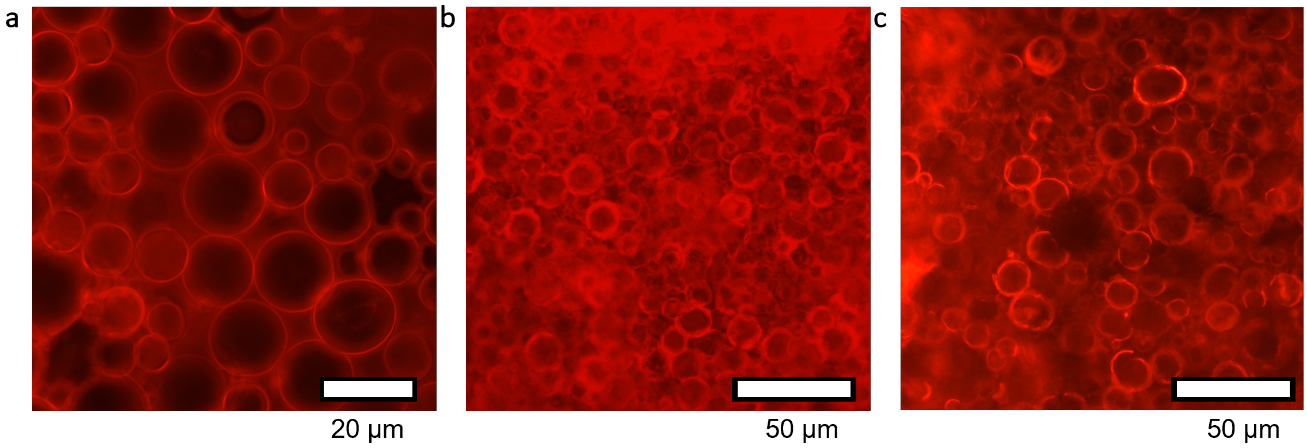

Figure S15. Fluorescence microscopy characterization of photocatalytic protocellular materials. a, Representative fluorescence microscopy image of a dispersed population of discrete PTA-CVs preloaded with $[Ru(bpy)_3]^{2+}$ (λ_{exc} = 405 nm, λ_{em} = 500-700 nm) in water. **b**, Representative fluorescence image of a central section of a protocellular sheet in PDDA solution (450-500 kDa, 20 wt% in water), assembled using $[Ru(bpy)_3]^{2+}$-loaded PTA-CVs. The image shows that the PCVs retain their original size, shape and structure, and are in close contact giving rise to localized small deformations. **c**, Representative fluorescence image of a central section of the protocellular sheet shown in (**b**) after complete transfer to water showing retention of the PCV structural integrity.

3. Supplementary Video

Video S1: Photocatalytic production of dioxygen bubbles by a 2D photocatalytic protocellular sheet

The video is composed of different clips showing the photocatalytic production of dioxygen bubbles by a 2D photocatalytic protocellular sheet immersed in 1.85 mL of Na_2SiF_6/$NaHCO_3$ buffer (3.8 mM in Na_2SiF_6 and 6.2 mM in $NaHCO_3$, pH 5.6) containing 40 µL of a solution of $Ru(bpy)_3Cl_2$ 50 mM and 100 µL of a solution of $Na_2S_2O_8$ 100 mM and irradiated with the brightfield microscope white light while being imaged.

4. References

1 Sartorel, A. *et al.* Polyoxometalate Embedding of a Tetraruthenium(IV)-oxo-core by Template-Directed Metalation of $[\gamma\text{-}SiW_{10}O_{36}]^{8-}$: A Totally Inorganic Oxygen-Evolving Catalyst. *Journal of the American Chemical Society* **130**, 5006-5007. DOI: 10.1021/ja077837f (2008).

2 Morgan, D. J. Resolving ruthenium: XPS studies of common ruthenium materials. *Surface and Interface Analysis* **47**, 1072-1079. DOI: 10.1002/sia.5852 (2015).

3 Anwar, N. *et al.* Surface Immobilization of a Tetra-Ruthenium Substituted Polyoxometalate Water Oxidation Catalyst Through the Employment of Conducting Polypyrrole and the Layer-by-Layer (LBL) Technique. *ACS Applied Materials & Interfaces* **6**, 8022-8031. DOI: 10.1021/am405295c (2014).
